# Supplementary figures and images for: Aurora kinase A promotes trained immunity via regulation of endogenous S-adenosylmethionine metabolism
Source: eLife. 2025 Sep 8;14:RP104138. doi: 10.7554/eLife.104138 (PMC12416886; doi:10.7554/eLife.104138)

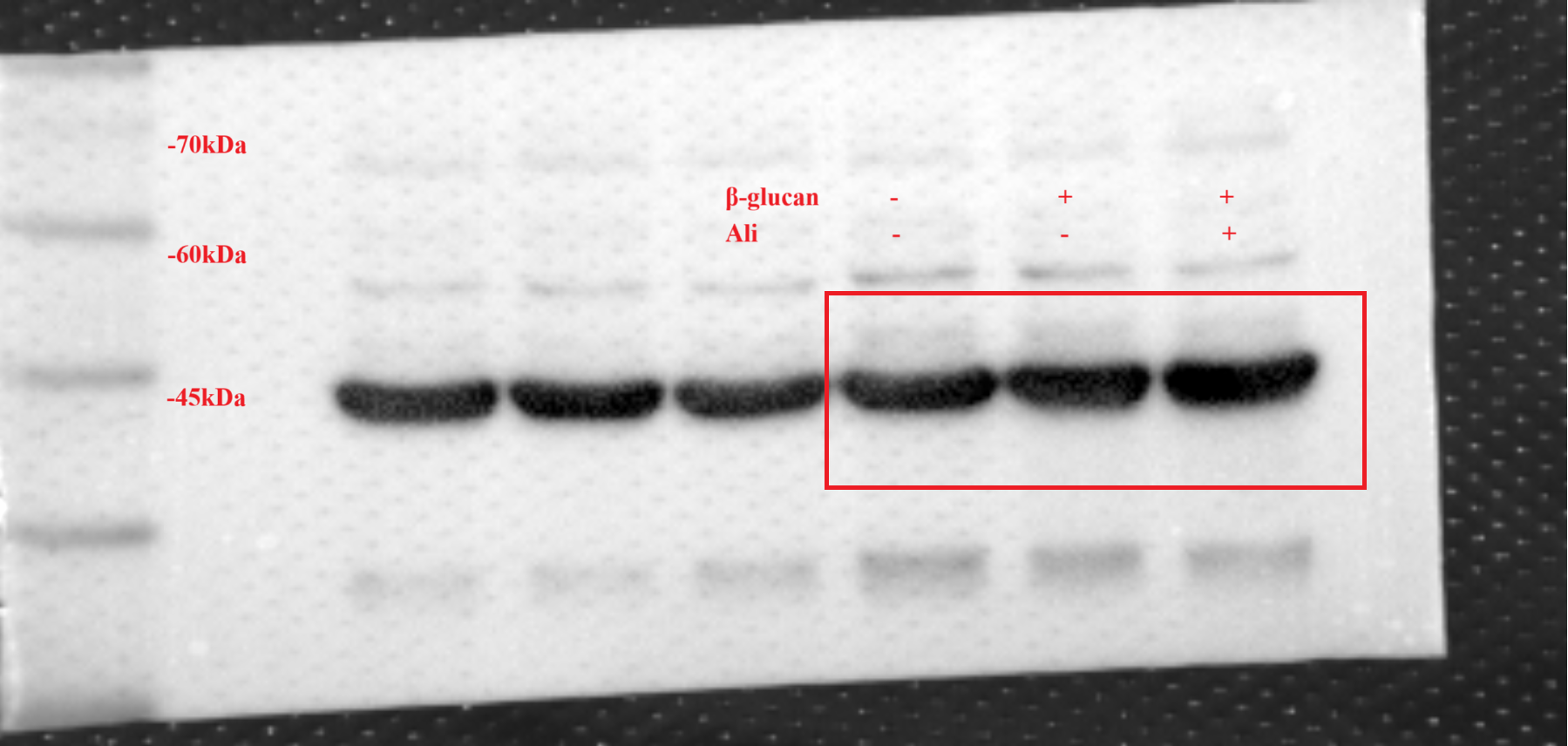

Supplement: Figure 1—source data 1. [file elife-104138-fig1-data1.zip › Figure 1_source Data 1/actin in 1D.tif]

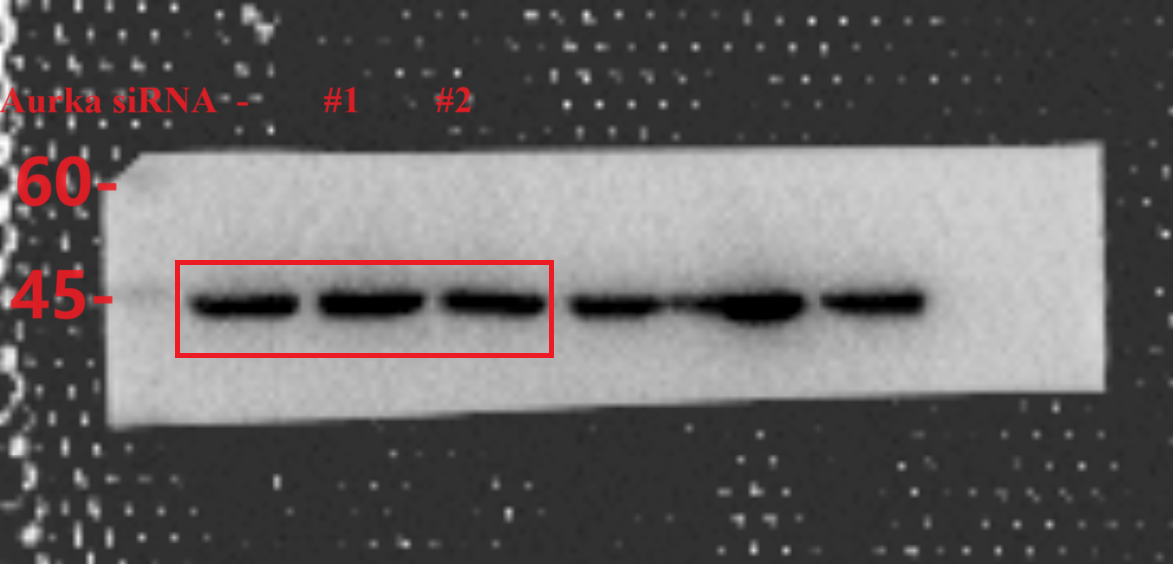

Supplement: Figure 1—source data 1. [file elife-104138-fig1-data1.zip › Figure 1_source Data 1/actin in 1E.tif]

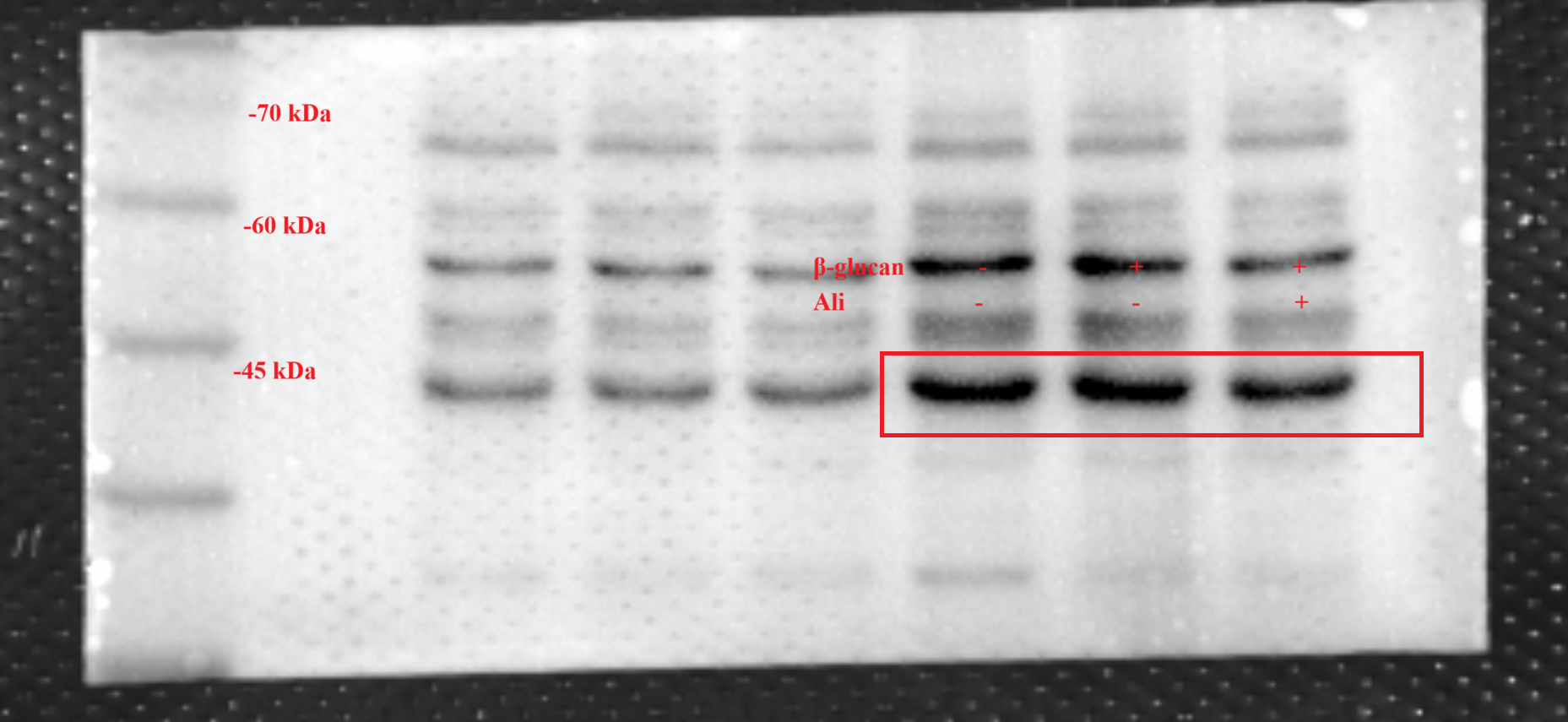

Supplement: Figure 1—source data 1. [file elife-104138-fig1-data1.zip › Figure 1_source Data 1/Aurora A in 1D.tif]

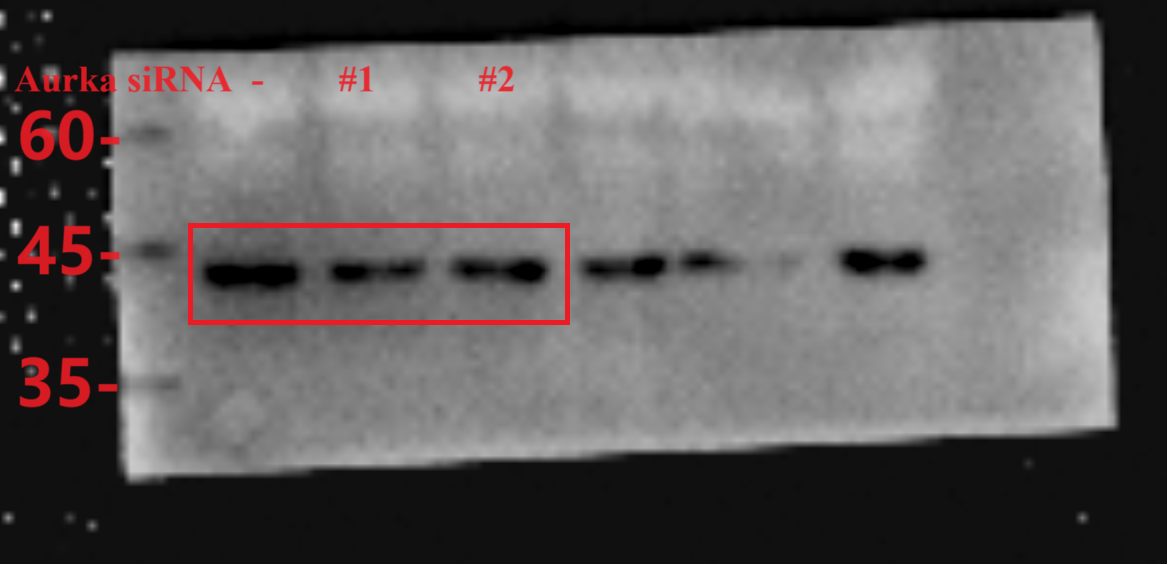

Supplement: Figure 1—source data 1. [file elife-104138-fig1-data1.zip › Figure 1_source Data 1/Aurora A in 1E.tif]

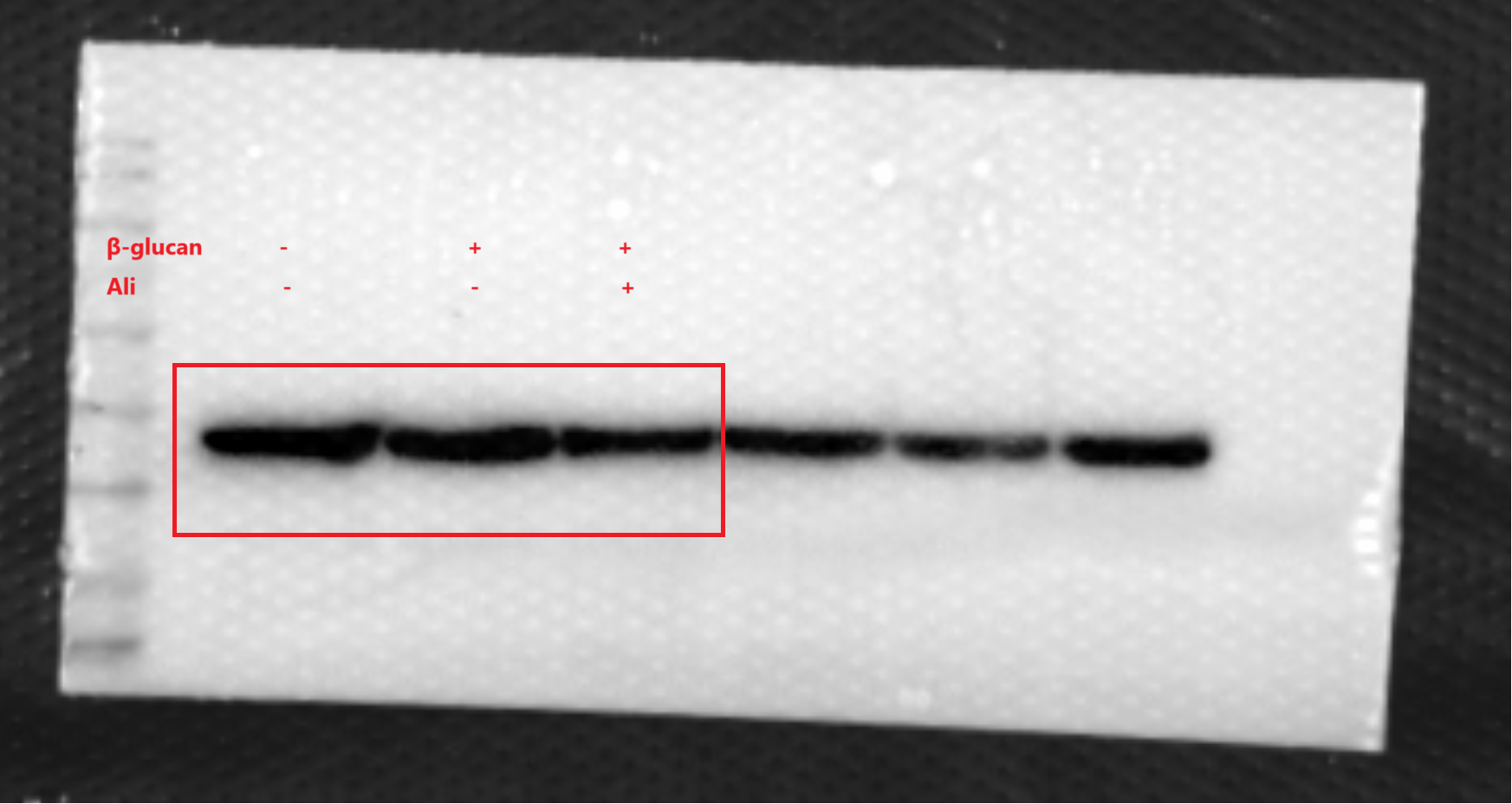

Supplement: Figure 1—source data 1. [file elife-104138-fig1-data1.zip › Figure 1_source Data 1/original 1D β-tubulin as control/actin in original 1D.tif]

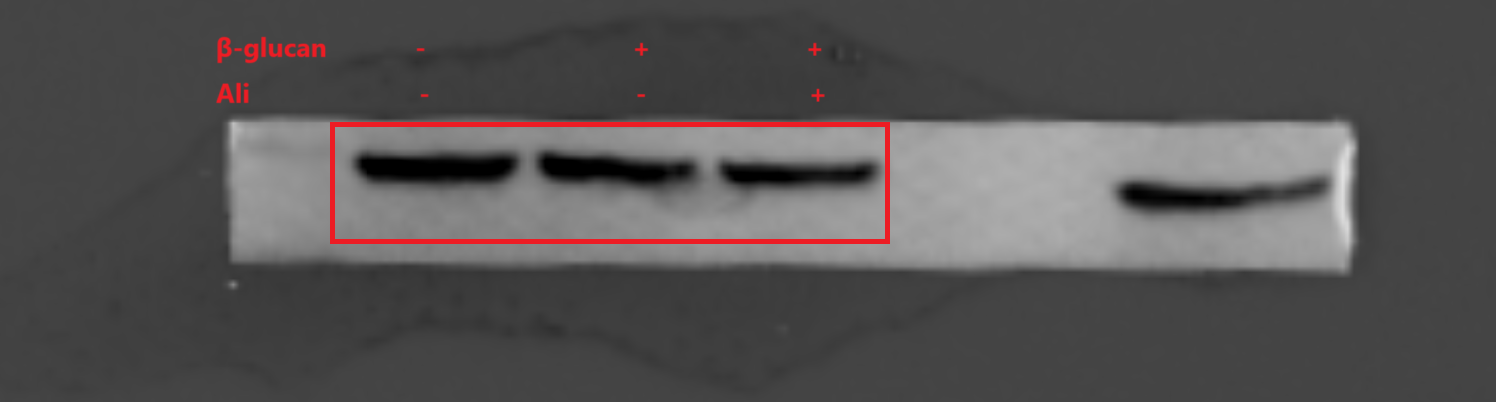

Supplement: Figure 1—source data 1. [file elife-104138-fig1-data1.zip › Figure 1_source Data 1/original 1D β-tubulin as control/Aurora A in original 1D.tif]

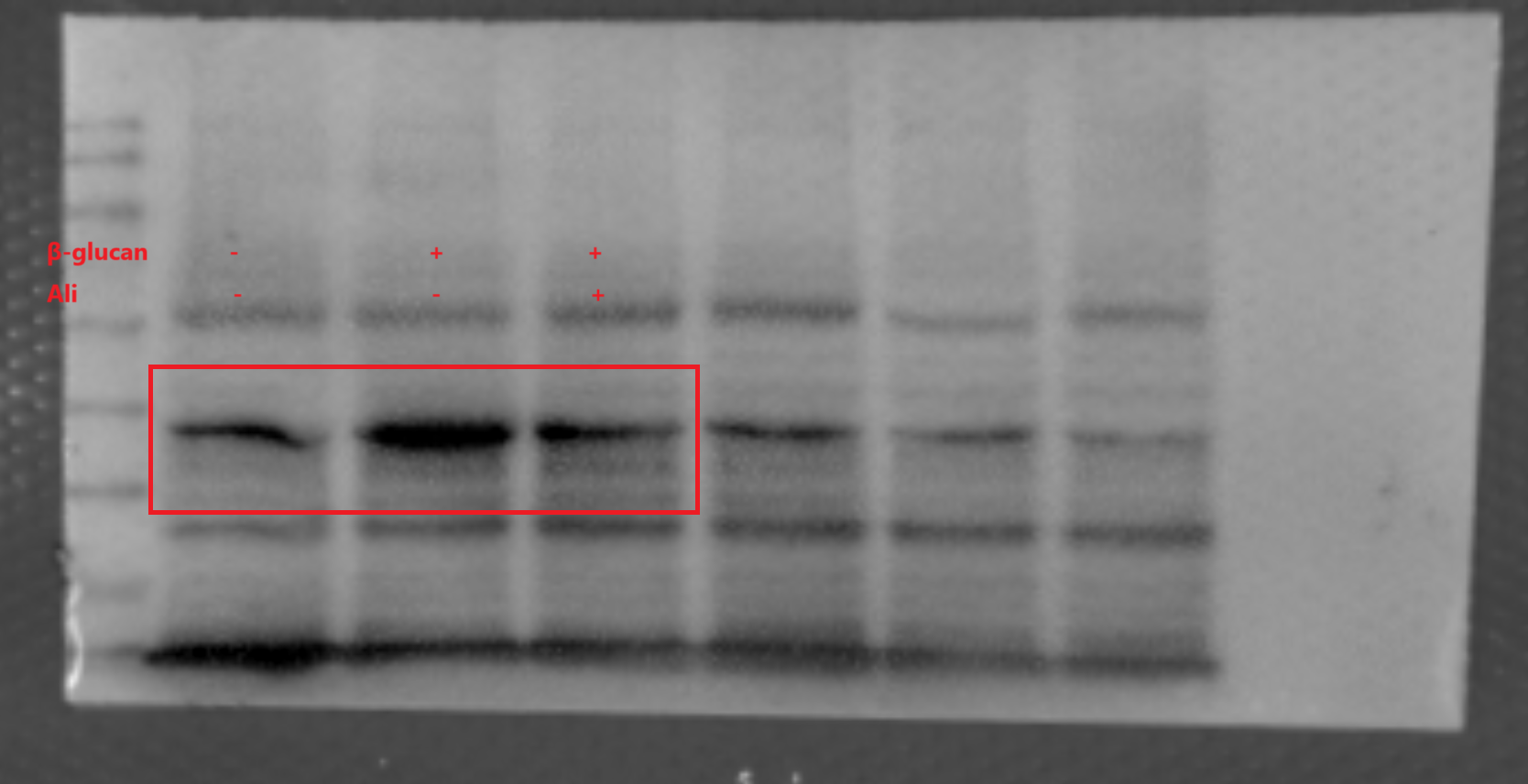

Supplement: Figure 1—source data 1. [file elife-104138-fig1-data1.zip › Figure 1_source Data 1/original 1D β-tubulin as control/phospho Aurora A in original 1D.tif]

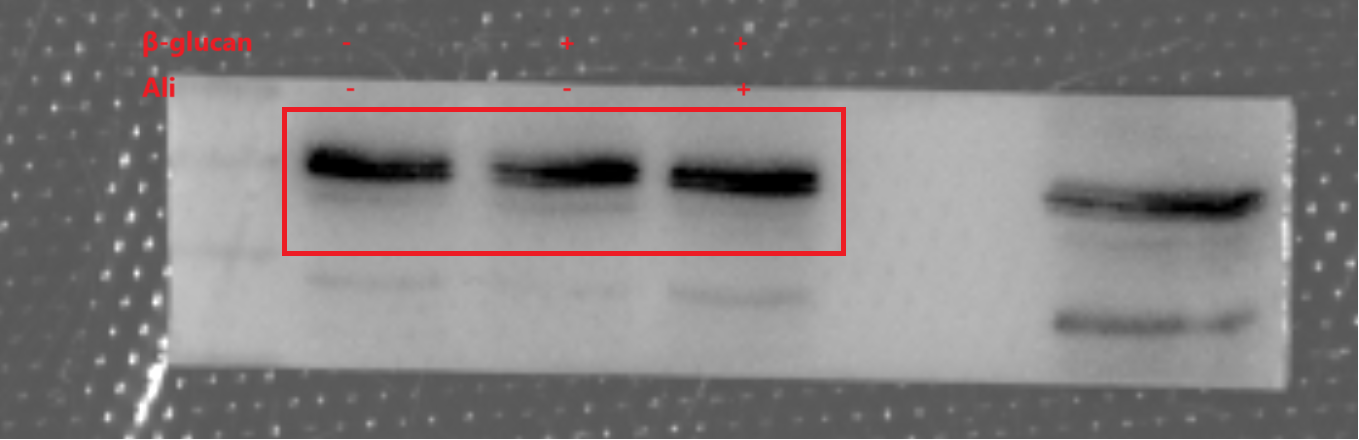

Supplement: Figure 1—source data 1. [file elife-104138-fig1-data1.zip › Figure 1_source Data 1/original 1D β-tubulin as control/β-tublin in original 1D.tif]

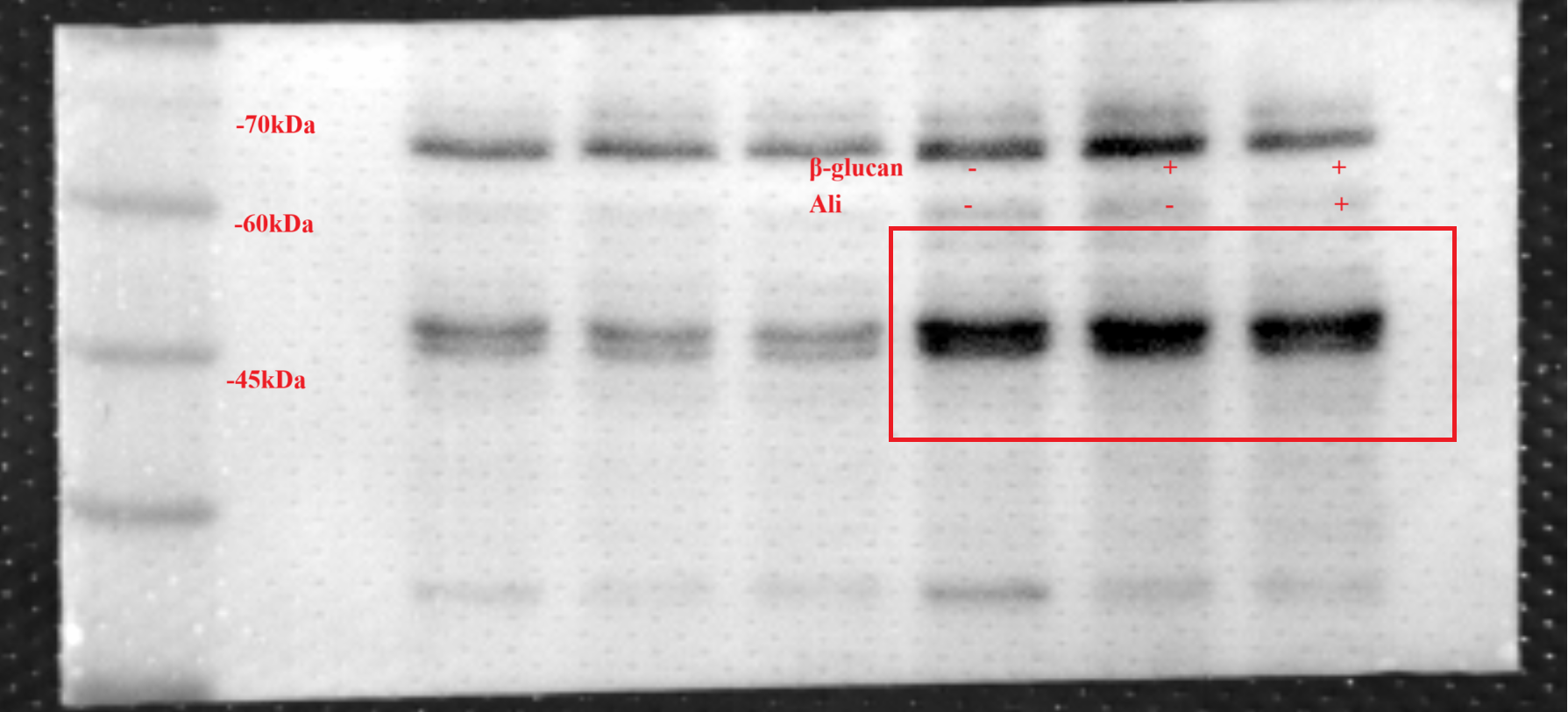

Supplement: Figure 1—source data 1. [file elife-104138-fig1-data1.zip › Figure 1_source Data 1/phos Aurora A in 1D.tif]

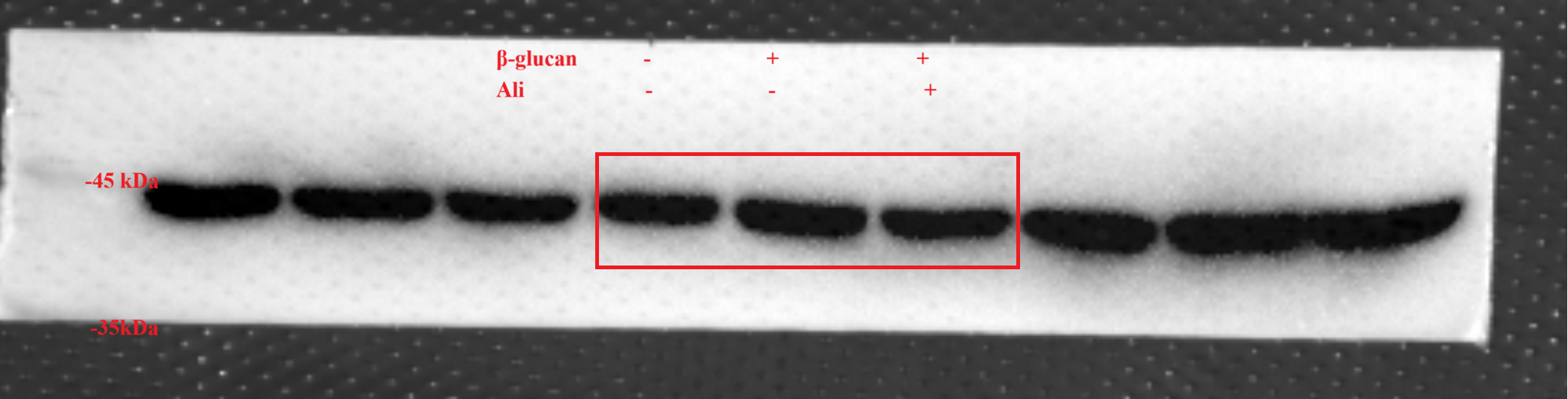

Supplement: Figure 1—source data 1. [file elife-104138-fig1-data1.zip › Figure 1_source Data 1/repeat 2 for 1D/actin in 1D.tif]

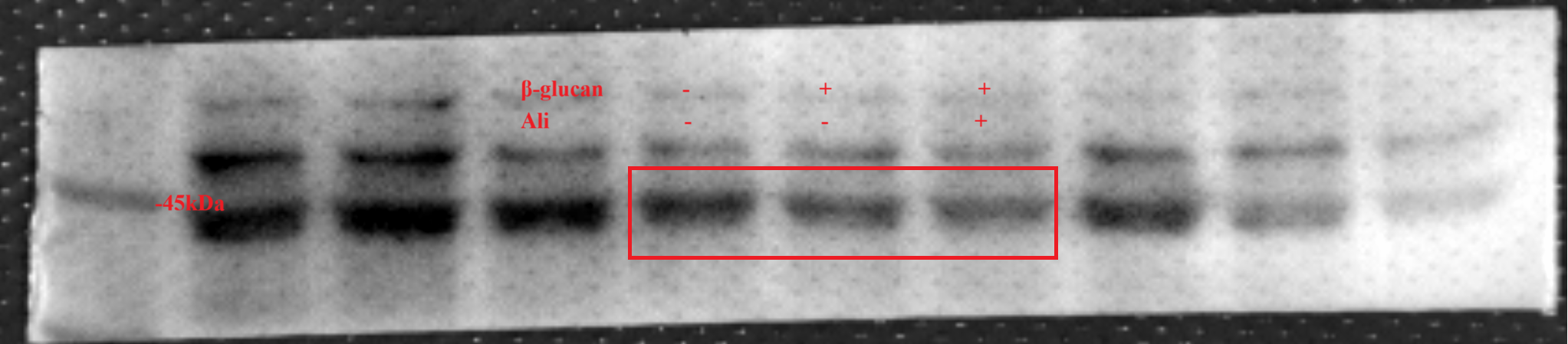

Supplement: Figure 1—source data 1. [file elife-104138-fig1-data1.zip › Figure 1_source Data 1/repeat 2 for 1D/Aurora A in 1D.tif]

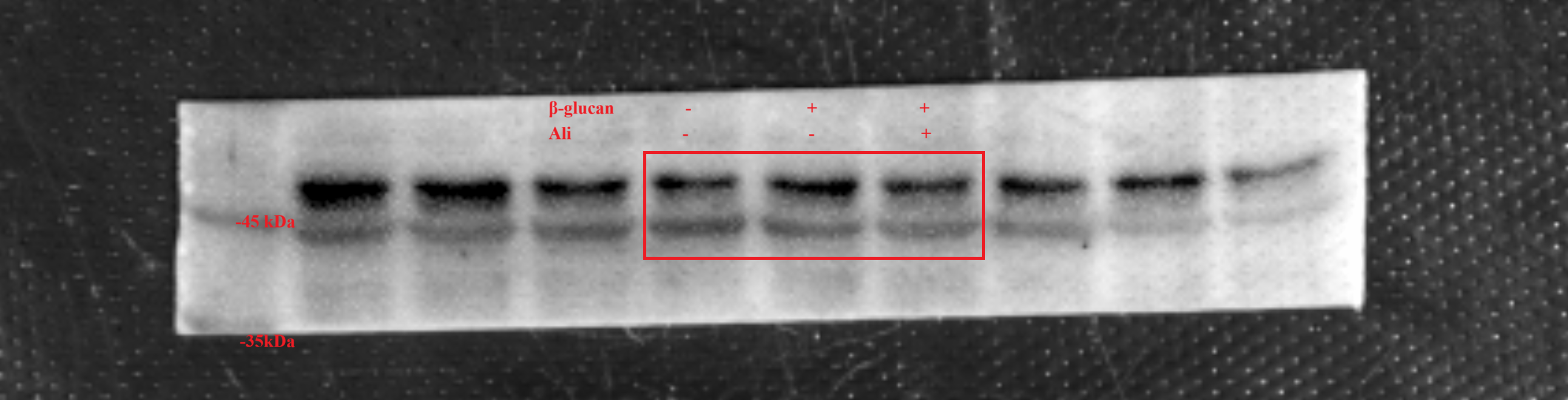

Supplement: Figure 1—source data 1. [file elife-104138-fig1-data1.zip › Figure 1_source Data 1/repeat 2 for 1D/phos Aurora A in 1D.tif]

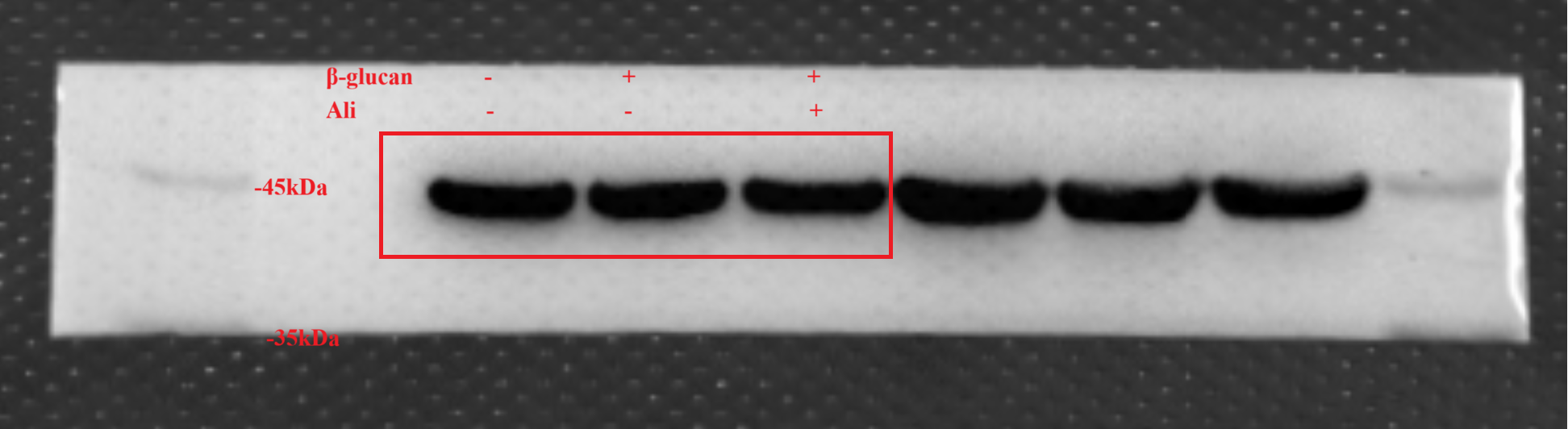

Supplement: Figure 1—source data 1. [file elife-104138-fig1-data1.zip › Figure 1_source Data 1/repeat 3 for 1D/actin in 1D.tif]

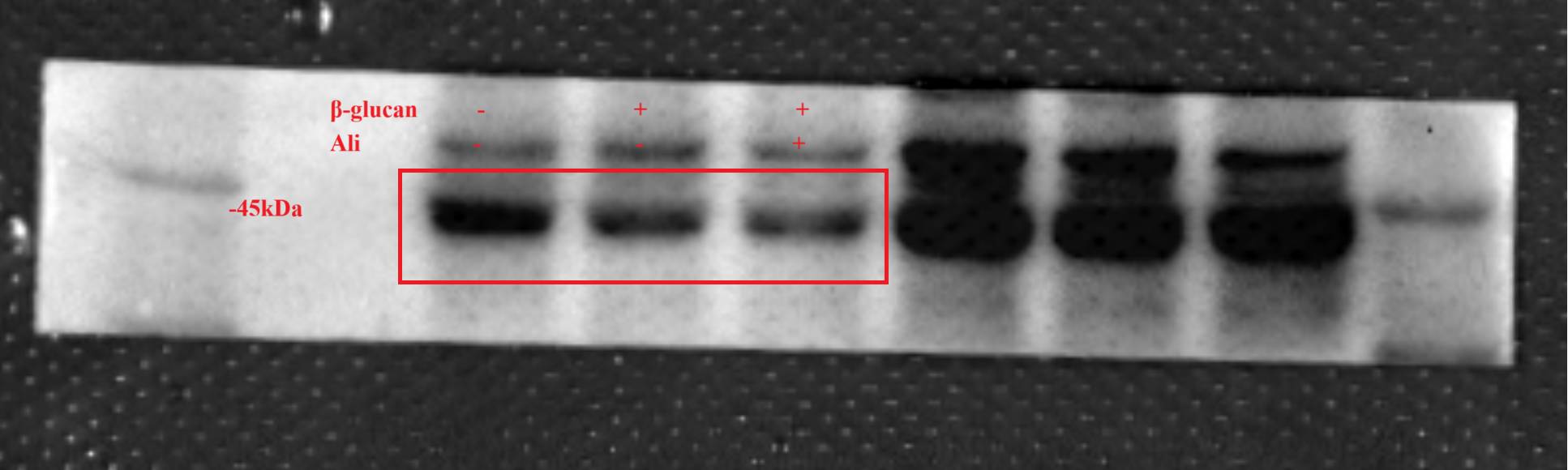

Supplement: Figure 1—source data 1. [file elife-104138-fig1-data1.zip › Figure 1_source Data 1/repeat 3 for 1D/Aurora A in 1D.tif]

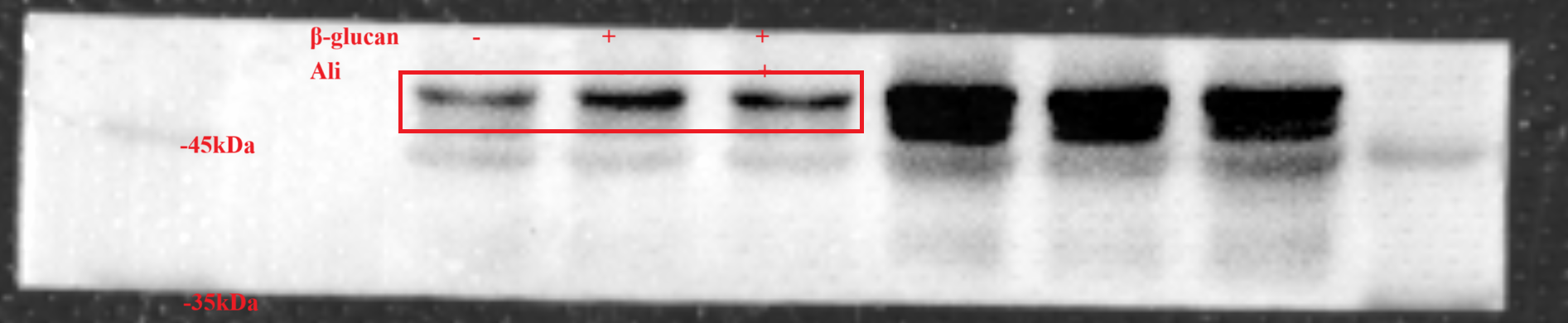

Supplement: Figure 1—source data 1. [file elife-104138-fig1-data1.zip › Figure 1_source Data 1/repeat 3 for 1D/phos Aurora A in 1D.tif]

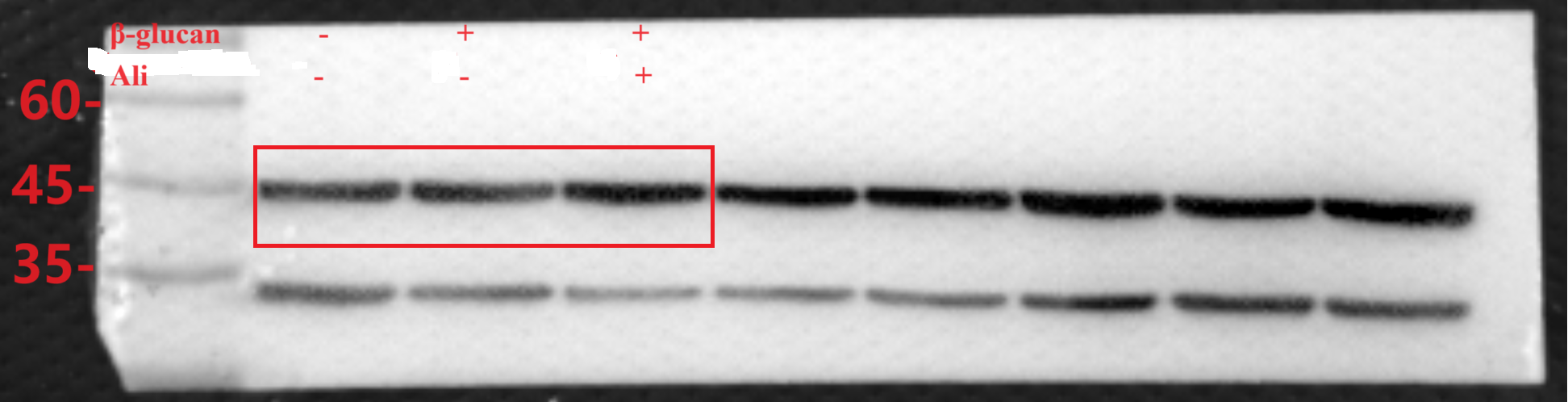

Supplement: Figure 3—source data 1. [file elife-104138-fig3-data1.zip › Figure 3_source Data 1/actin in 3H.tif]

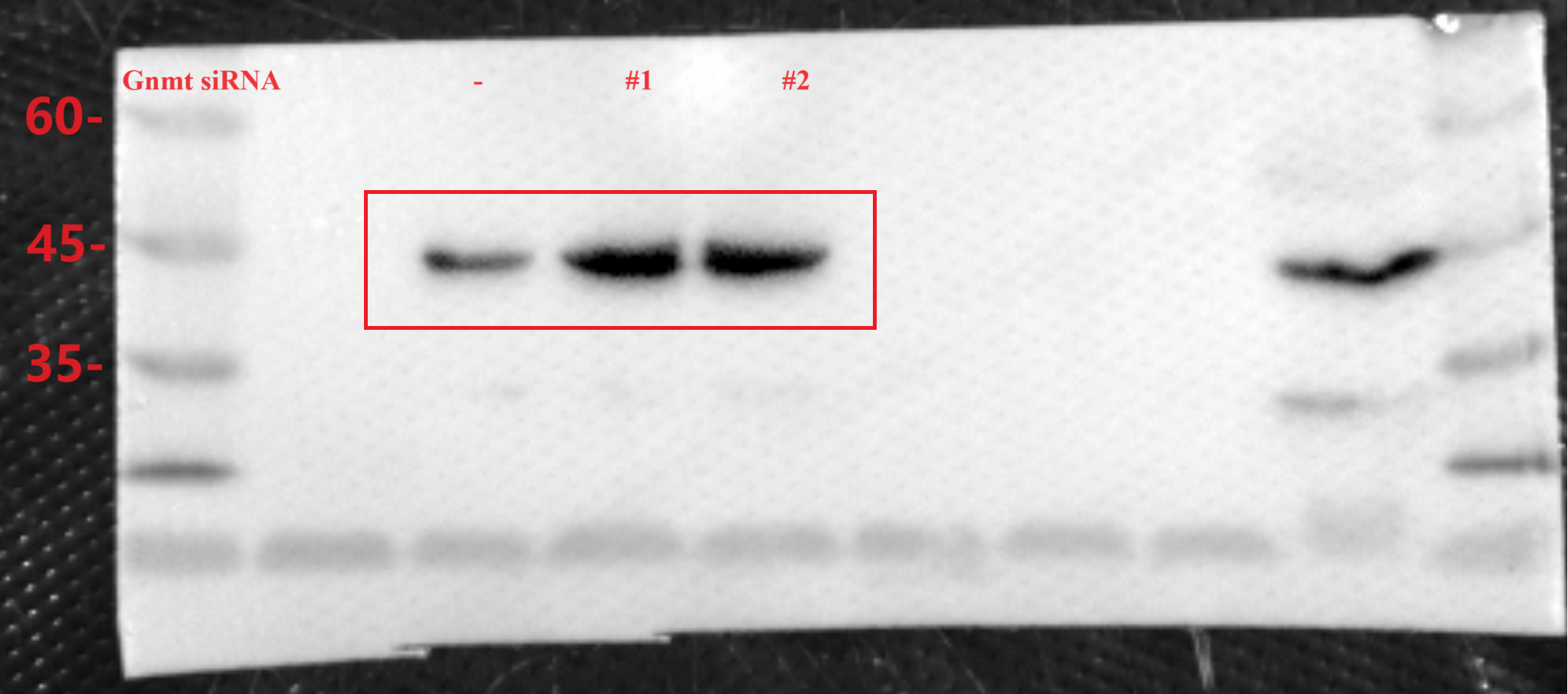

Supplement: Figure 3—source data 1. [file elife-104138-fig3-data1.zip › Figure 3_source Data 1/actin in 3I.tif]

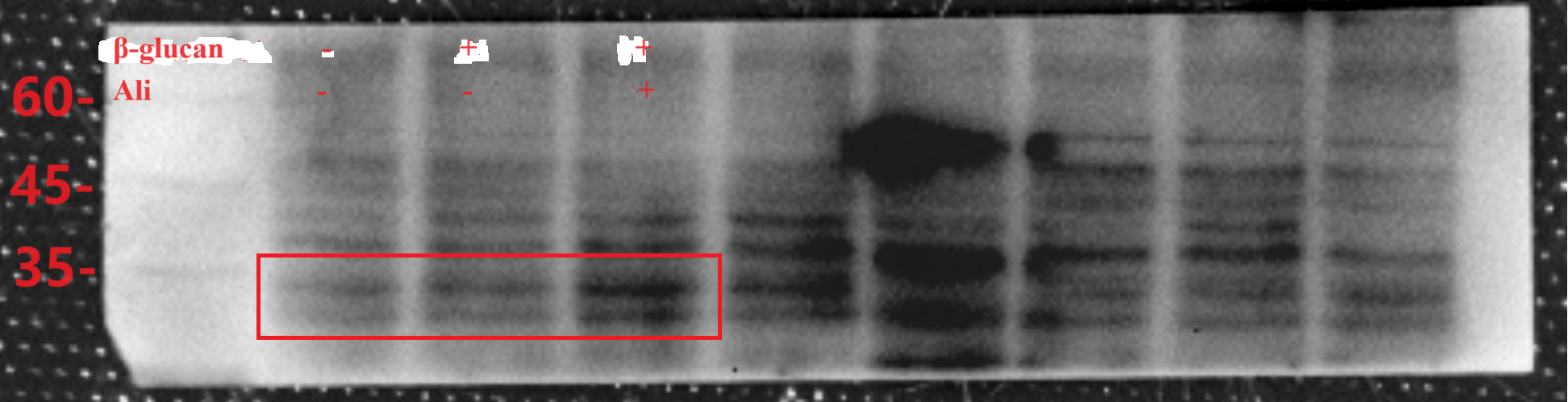

Supplement: Figure 3—source data 1. [file elife-104138-fig3-data1.zip › Figure 3_source Data 1/GNMT in 3H.tif]

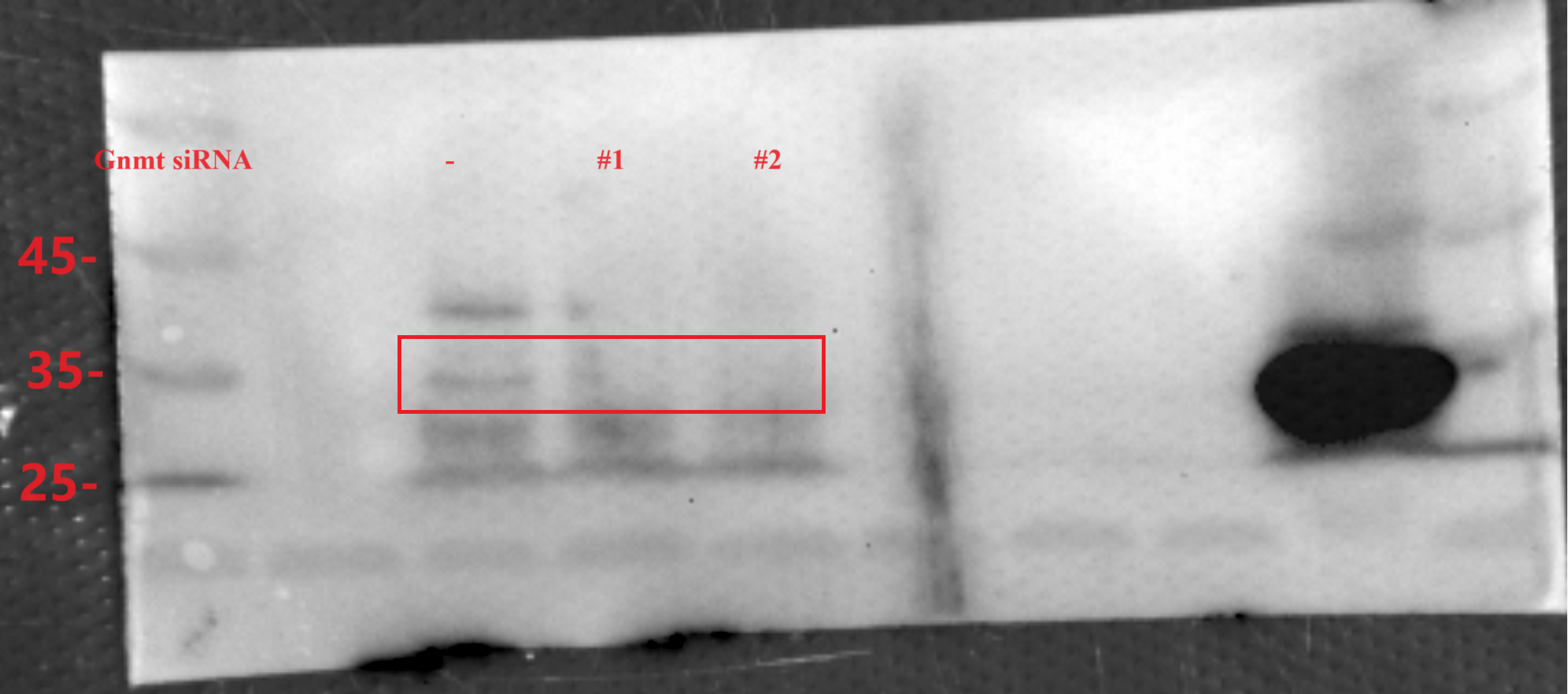

Supplement: Figure 3—source data 1. [file elife-104138-fig3-data1.zip › Figure 3_source Data 1/GNMT in 3I.tif]

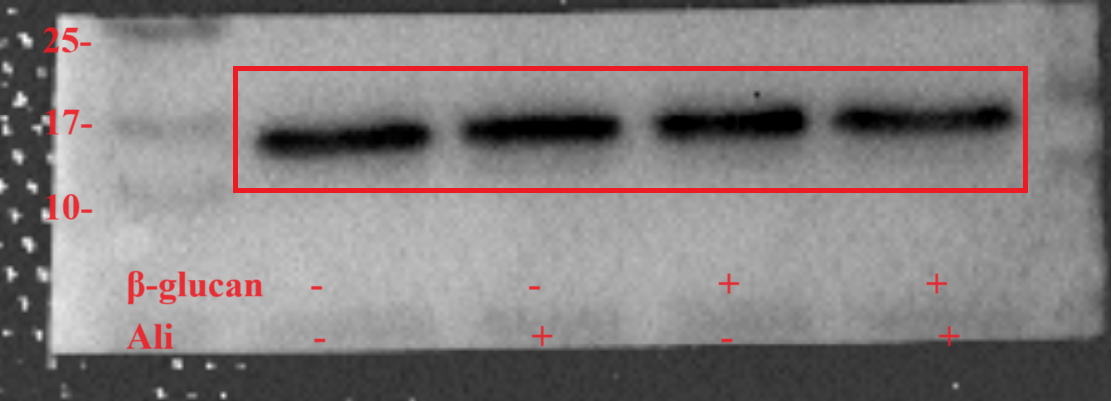

Supplement: Figure 4—source data 1. [file elife-104138-fig4-data1.zip › Figure 4_source Data 1/H3 in 4A.tif]

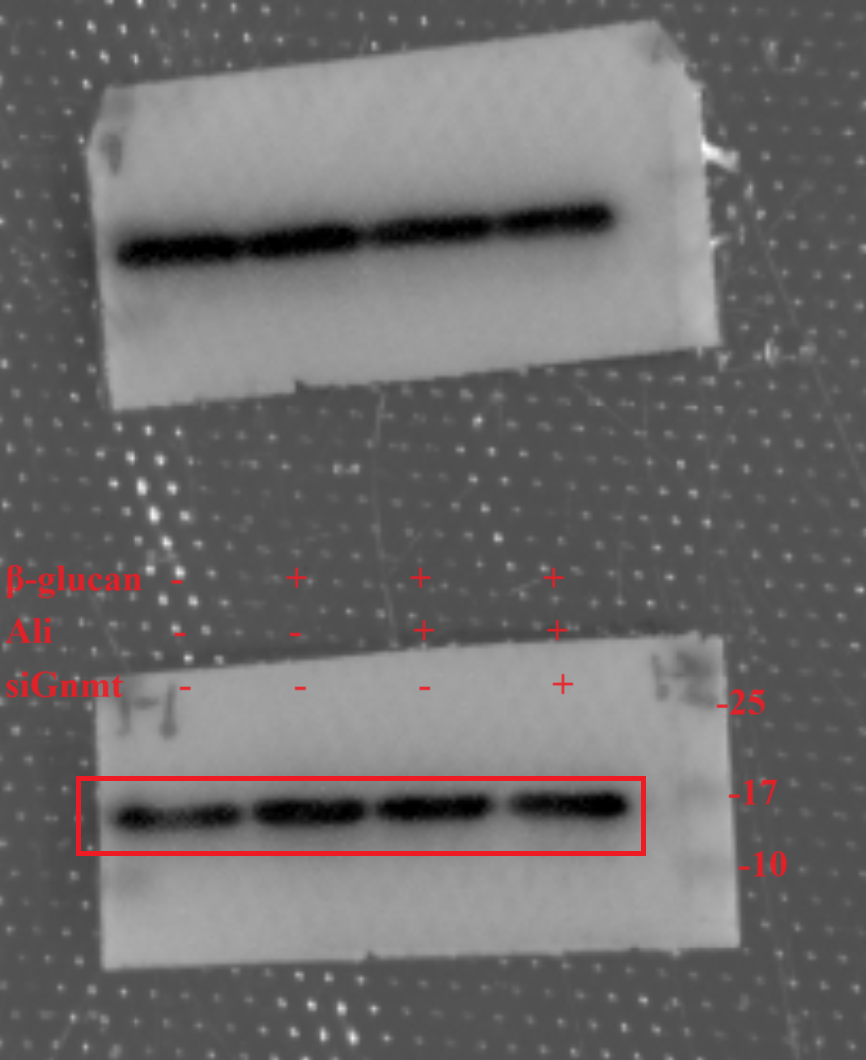

Supplement: Figure 4—source data 1. [file elife-104138-fig4-data1.zip › Figure 4_source Data 1/H3 in 4C.tif]

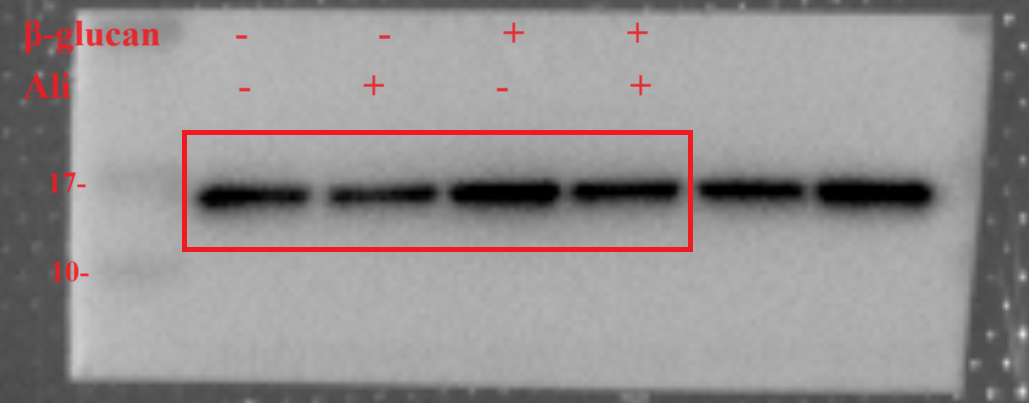

Supplement: Figure 4—source data 1. [file elife-104138-fig4-data1.zip › Figure 4_source Data 1/H3K27me3 in 4A.tif]

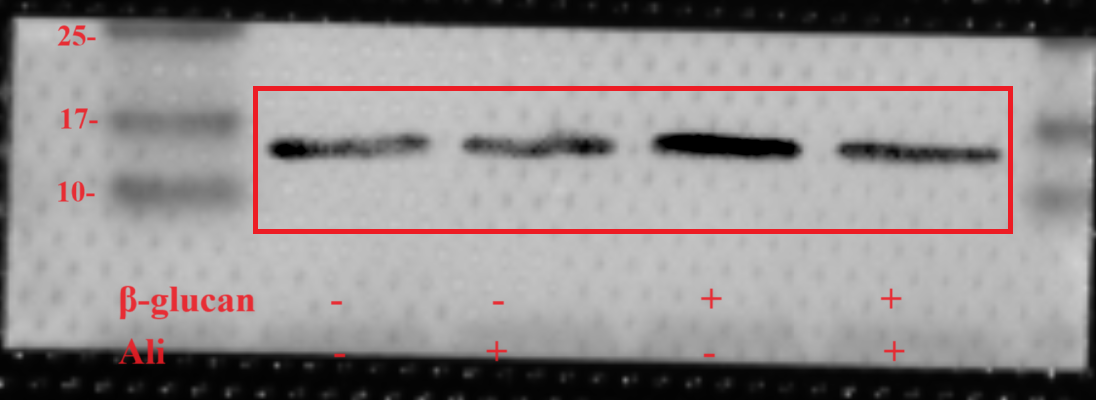

Supplement: Figure 4—source data 1. [file elife-104138-fig4-data1.zip › Figure 4_source Data 1/H3K36me3 in 4A.tif]

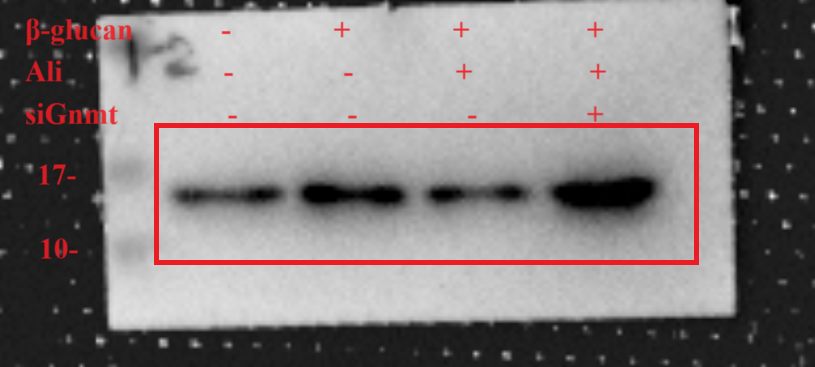

Supplement: Figure 4—source data 1. [file elife-104138-fig4-data1.zip › Figure 4_source Data 1/H3K36me3 in 4C.tif]

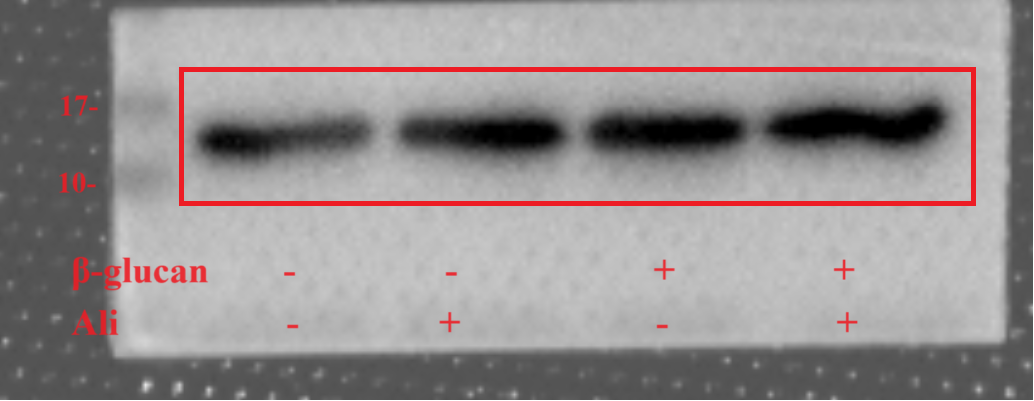

Supplement: Figure 4—source data 1. [file elife-104138-fig4-data1.zip › Figure 4_source Data 1/H3K4me1 in 4A.tif]

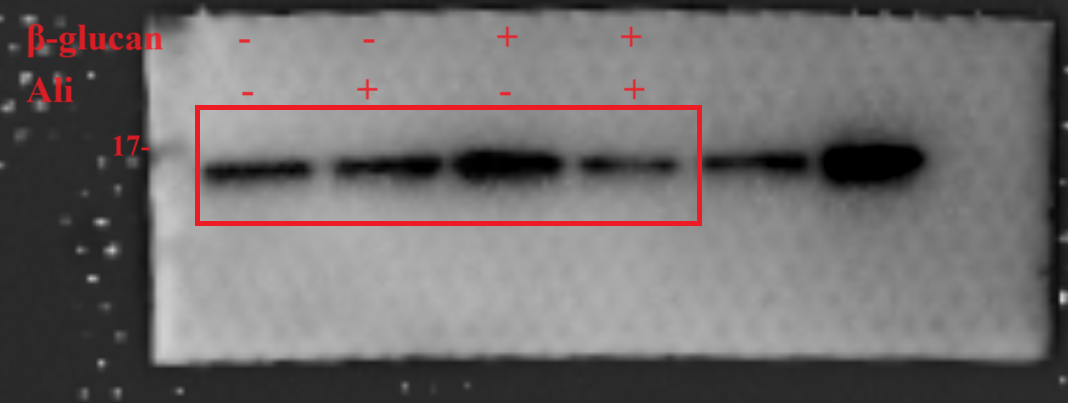

Supplement: Figure 4—source data 1. [file elife-104138-fig4-data1.zip › Figure 4_source Data 1/H3K4me3 in 4A.tif]

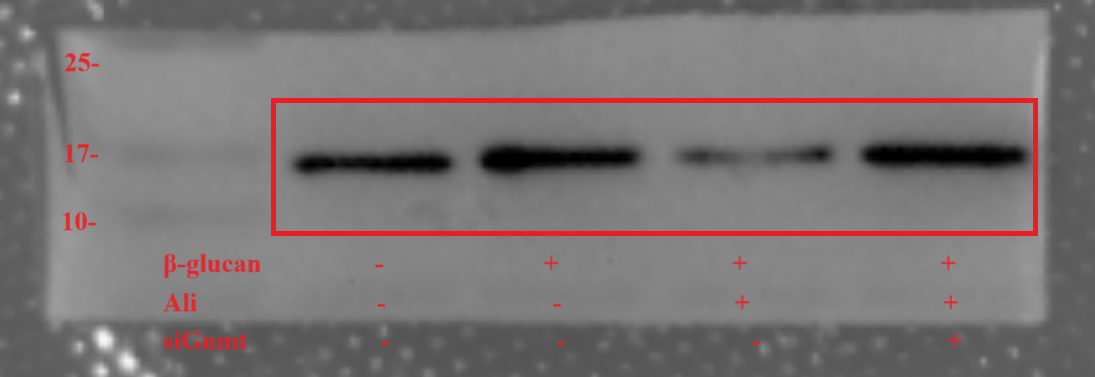

Supplement: Figure 4—source data 1. [file elife-104138-fig4-data1.zip › Figure 4_source Data 1/H3K4me3 in 4C.tif]

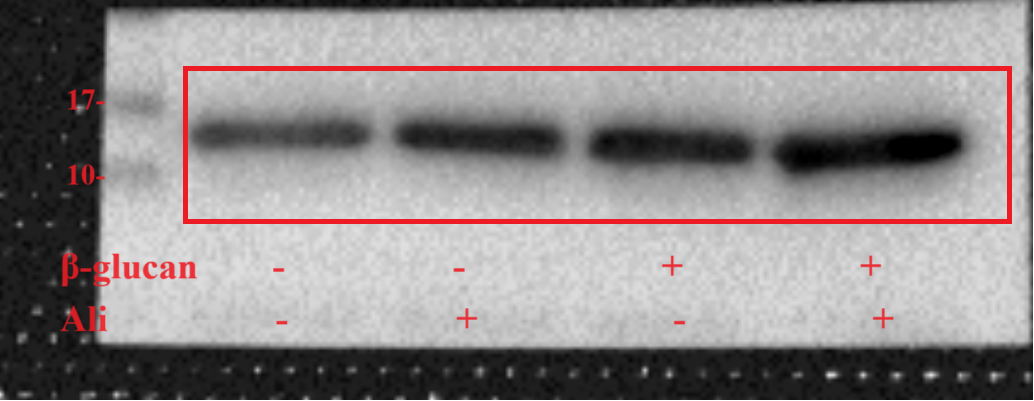

Supplement: Figure 4—source data 1. [file elife-104138-fig4-data1.zip › Figure 4_source Data 1/H3K9me3 in 4A.tif]

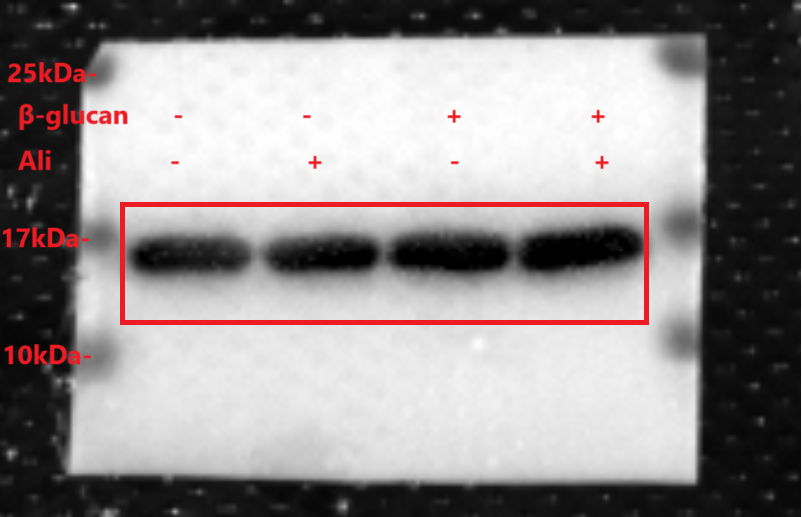

Supplement: Figure 4—source data 1. [file elife-104138-fig4-data1.zip › Figure 4_source Data 1/rep2/H3 in 4A.tif]

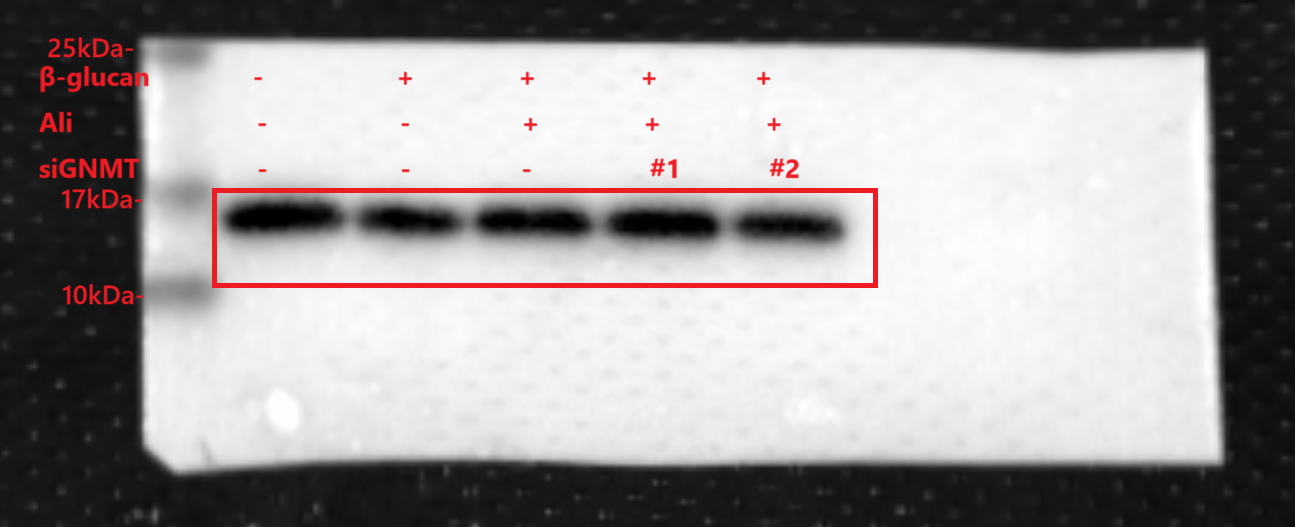

Supplement: Figure 4—source data 1. [file elife-104138-fig4-data1.zip › Figure 4_source Data 1/rep2/H3 in 4C.tif]

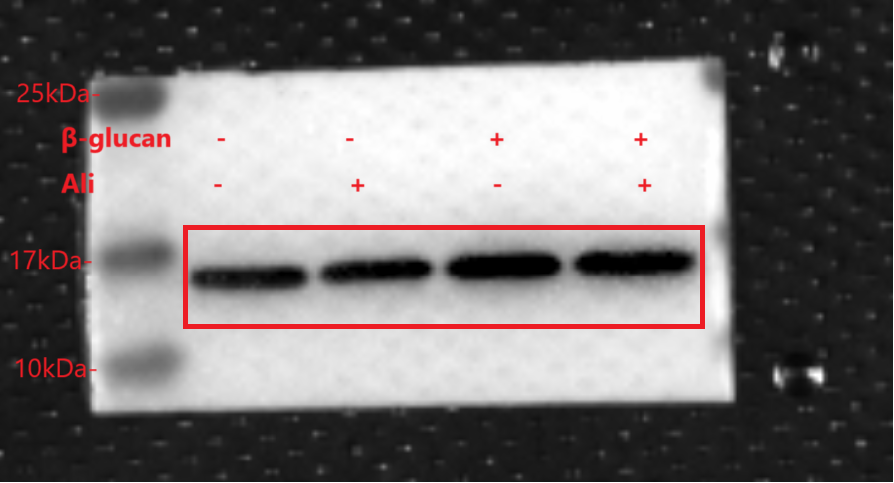

Supplement: Figure 4—source data 1. [file elife-104138-fig4-data1.zip › Figure 4_source Data 1/rep2/H3K27me3 in 4A.tif]

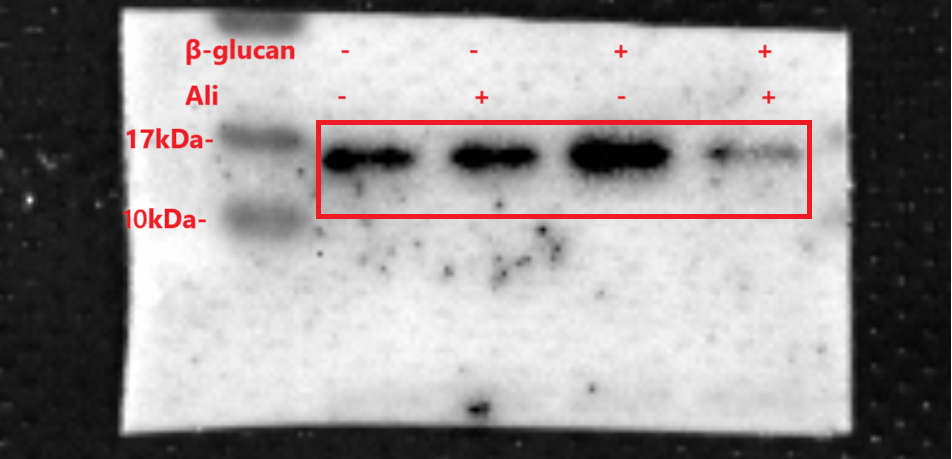

Supplement: Figure 4—source data 1. [file elife-104138-fig4-data1.zip › Figure 4_source Data 1/rep2/H3K36me3 in 4A.tif]

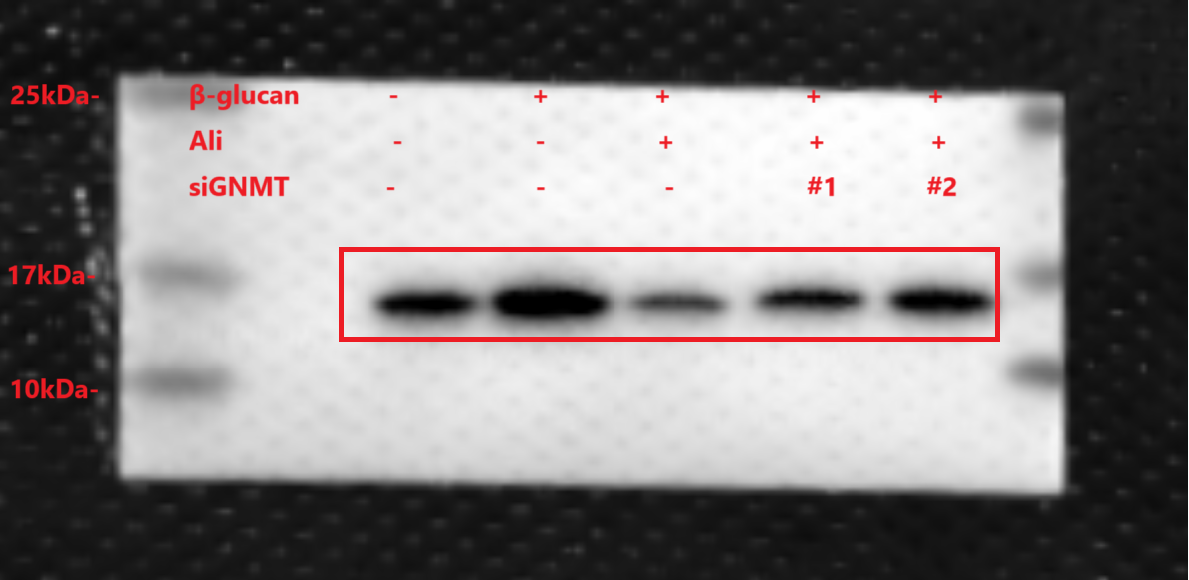

Supplement: Figure 4—source data 1. [file elife-104138-fig4-data1.zip › Figure 4_source Data 1/rep2/H3K36me3 in 4C.tif]

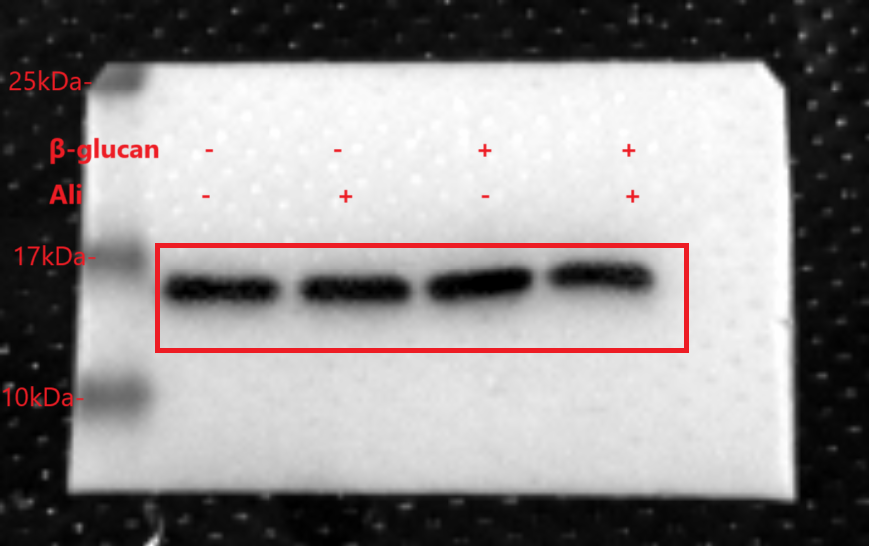

Supplement: Figure 4—source data 1. [file elife-104138-fig4-data1.zip › Figure 4_source Data 1/rep2/H3K4me1 in 4A.tif]

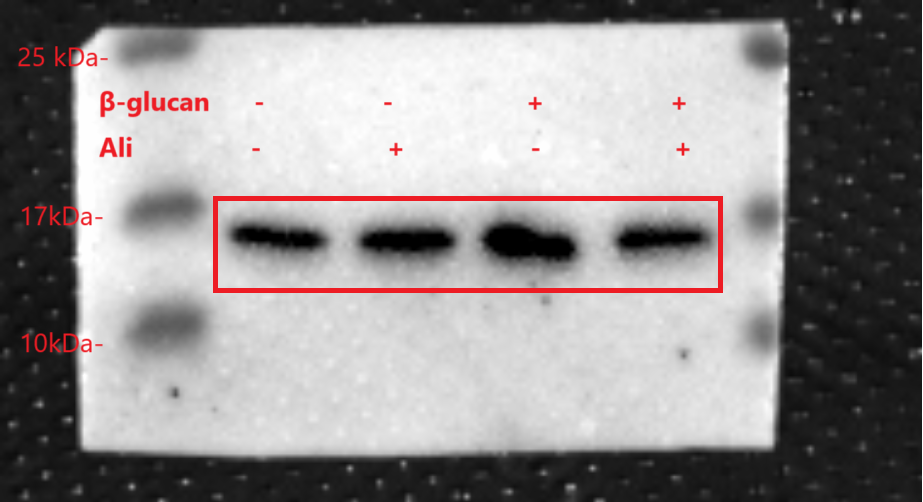

Supplement: Figure 4—source data 1. [file elife-104138-fig4-data1.zip › Figure 4_source Data 1/rep2/H3K4me3 in 4A.tif]

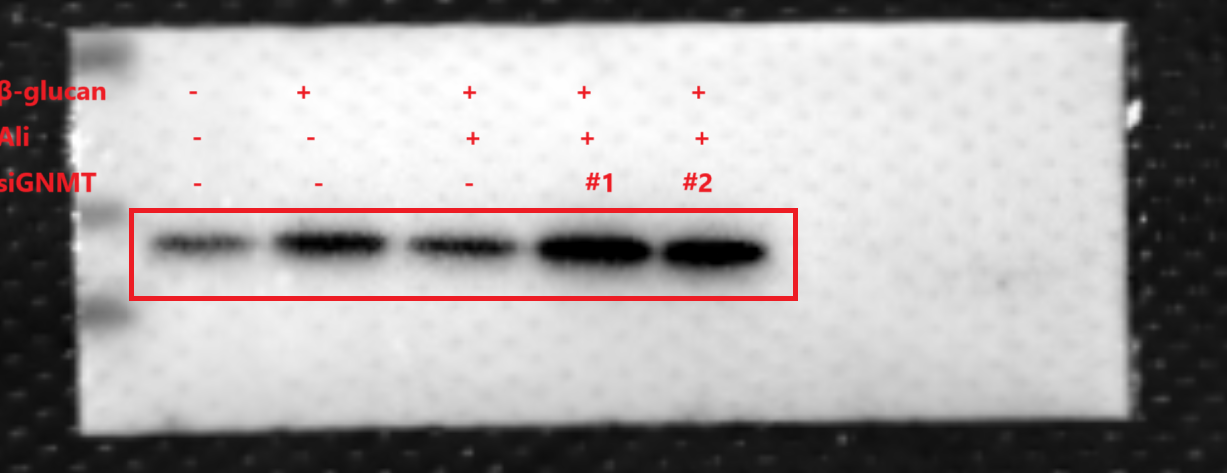

Supplement: Figure 4—source data 1. [file elife-104138-fig4-data1.zip › Figure 4_source Data 1/rep2/H3K4me3 in 4C.tif]

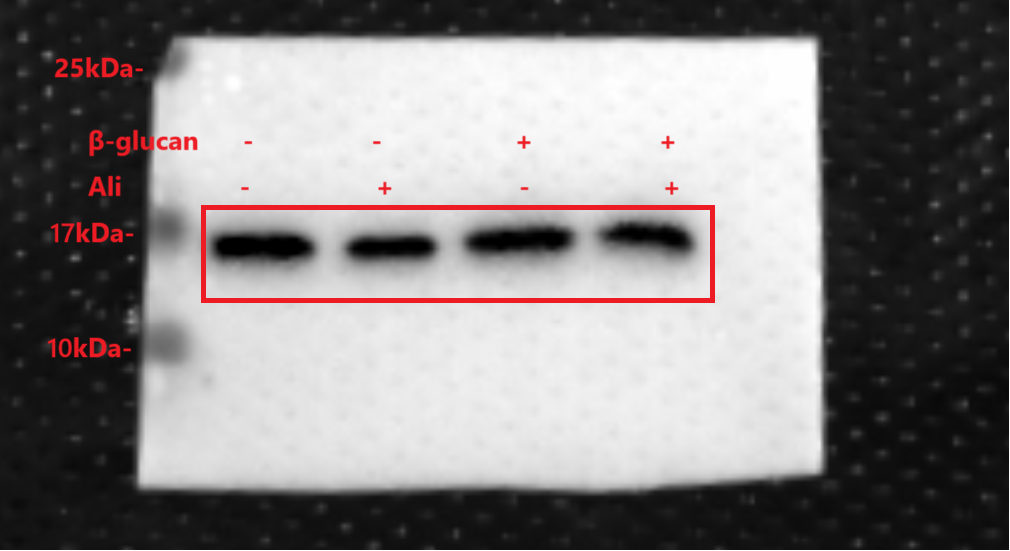

Supplement: Figure 4—source data 1. [file elife-104138-fig4-data1.zip › Figure 4_source Data 1/rep2/H3K9me3 in 4A.tif]

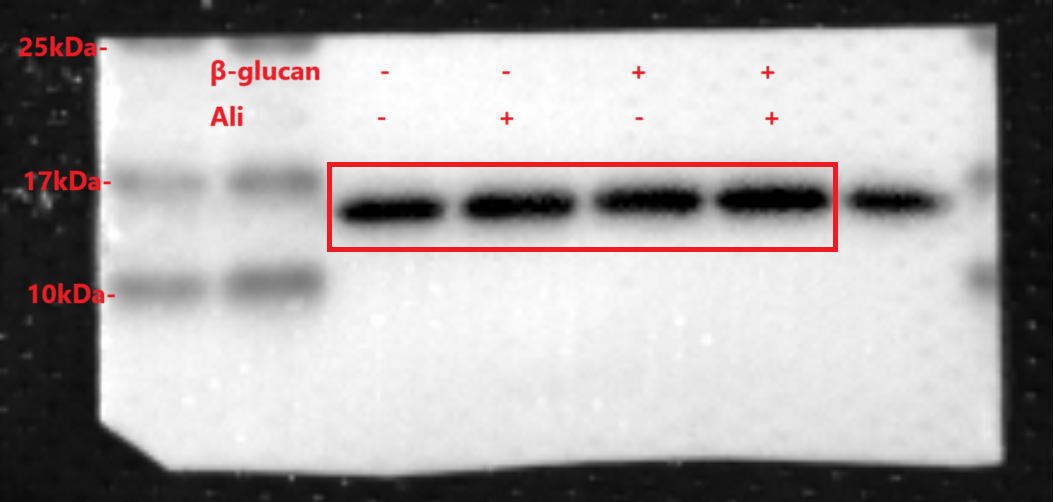

Supplement: Figure 4—source data 1. [file elife-104138-fig4-data1.zip › Figure 4_source Data 1/rep3/H3 in 4A.tif]

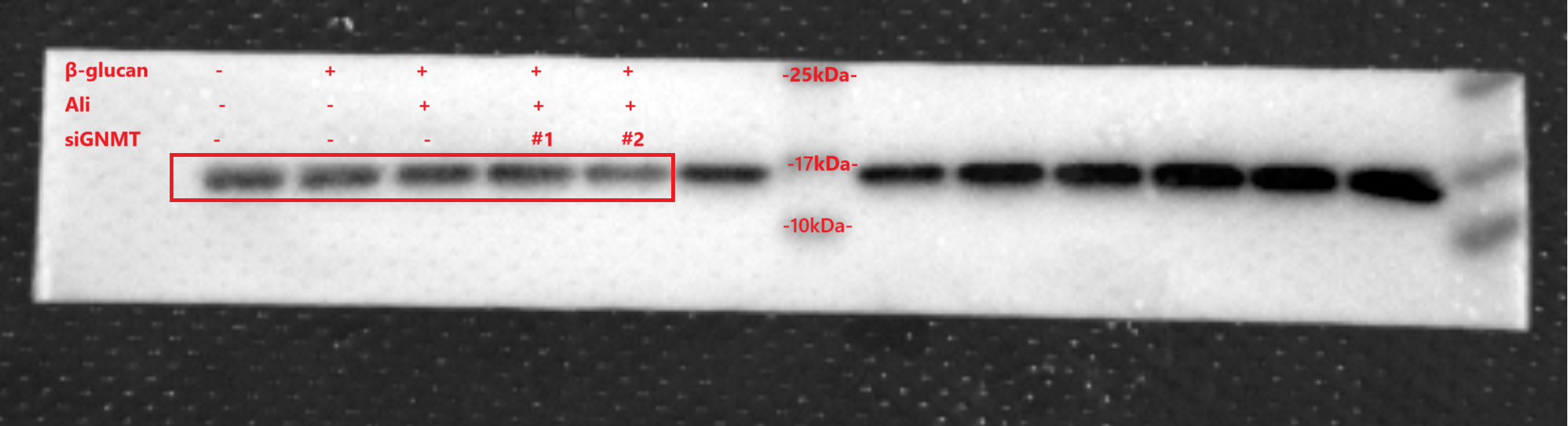

Supplement: Figure 4—source data 1. [file elife-104138-fig4-data1.zip › Figure 4_source Data 1/rep3/H3 in 4C.tif]

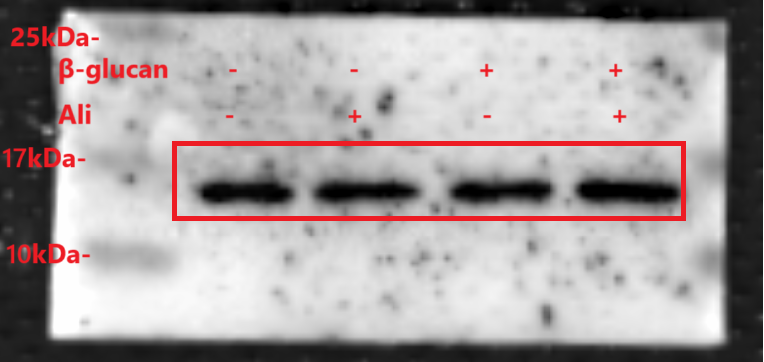

Supplement: Figure 4—source data 1. [file elife-104138-fig4-data1.zip › Figure 4_source Data 1/rep3/H3K27me3 in 4A.tif]

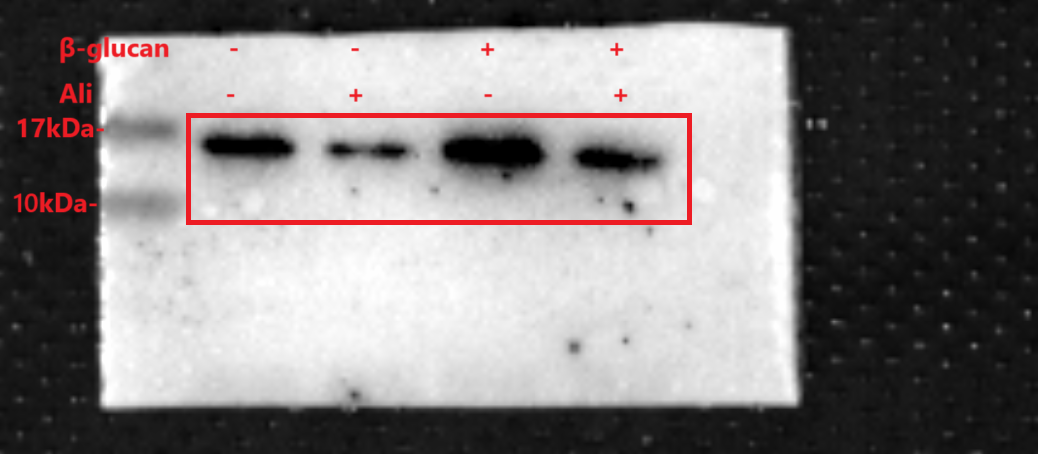

Supplement: Figure 4—source data 1. [file elife-104138-fig4-data1.zip › Figure 4_source Data 1/rep3/H3K36me3 in 4A.tif]

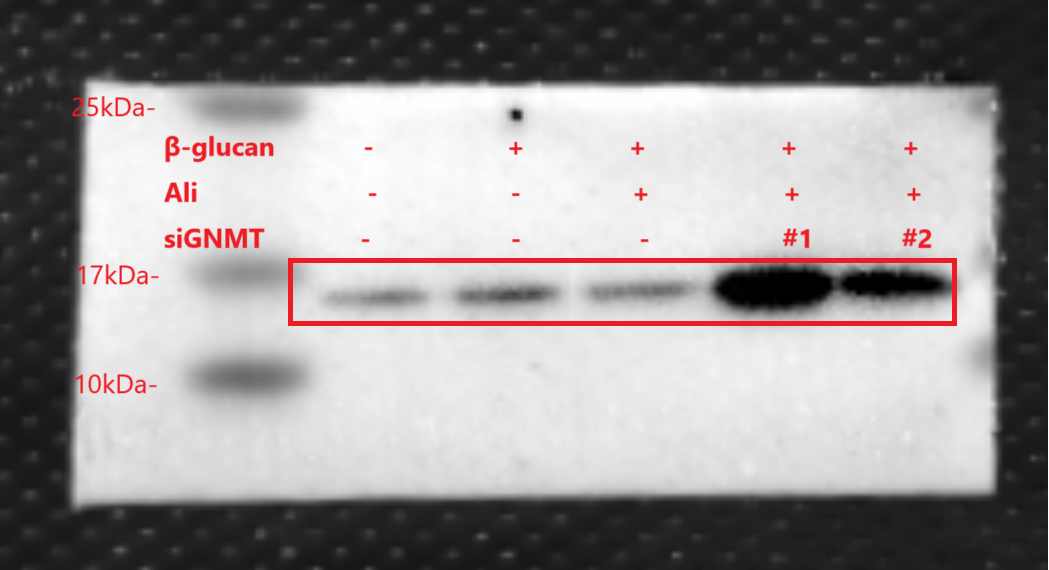

Supplement: Figure 4—source data 1. [file elife-104138-fig4-data1.zip › Figure 4_source Data 1/rep3/H3K36me3 in 4C.tif]

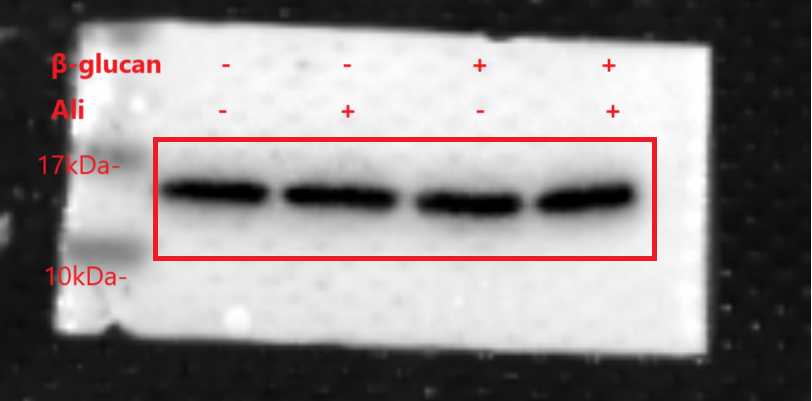

Supplement: Figure 4—source data 1. [file elife-104138-fig4-data1.zip › Figure 4_source Data 1/rep3/H3K4me1 in 4A.tif]

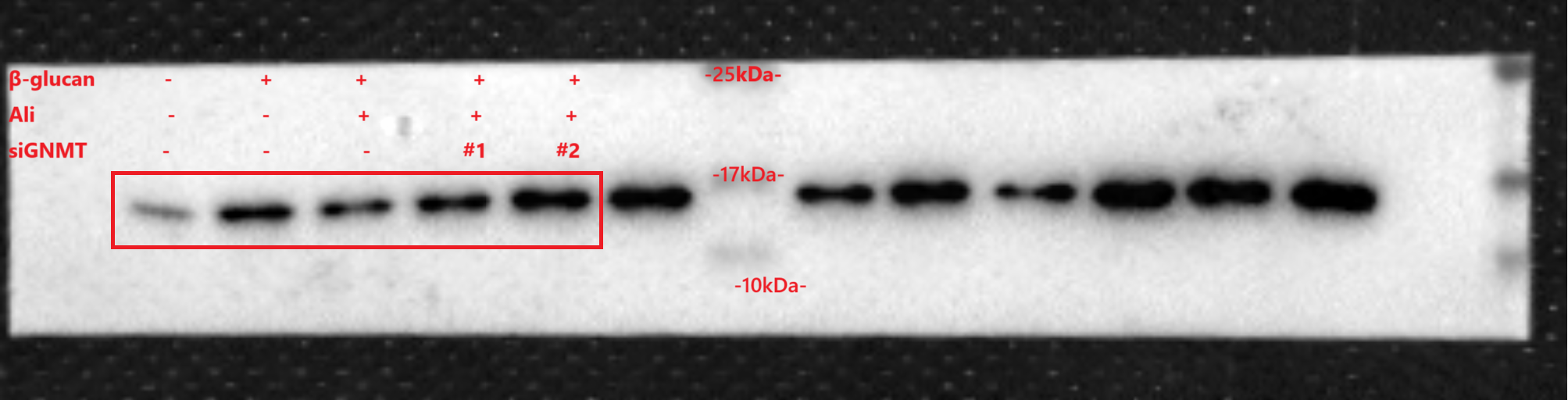

Supplement: Figure 4—source data 1. [file elife-104138-fig4-data1.zip › Figure 4_source Data 1/rep3/H3K4me3 in 4C.tif]

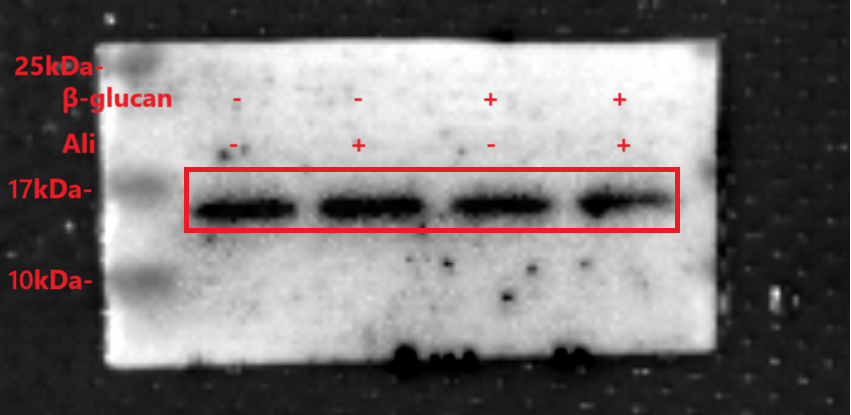

Supplement: Figure 4—source data 1. [file elife-104138-fig4-data1.zip › Figure 4_source Data 1/rep3/H3K9me3 in 4A.tif]

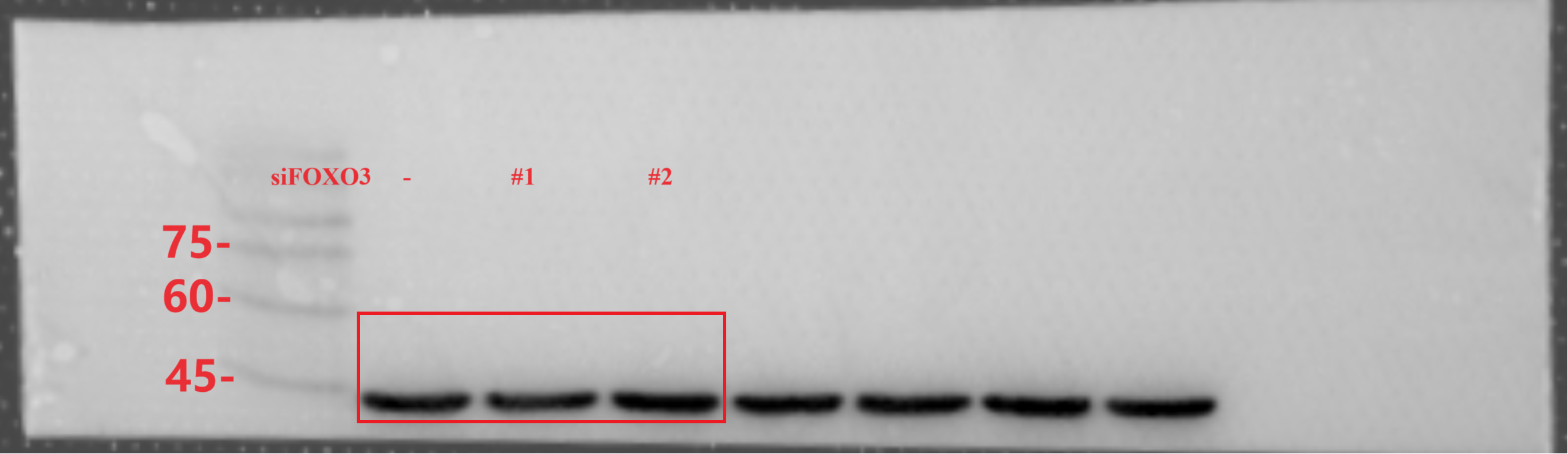

Supplement: Figure 5—source data 1. [file elife-104138-fig5-data1.zip › Figure 5_source Data 1/Figure 5A/5A actin.tif]

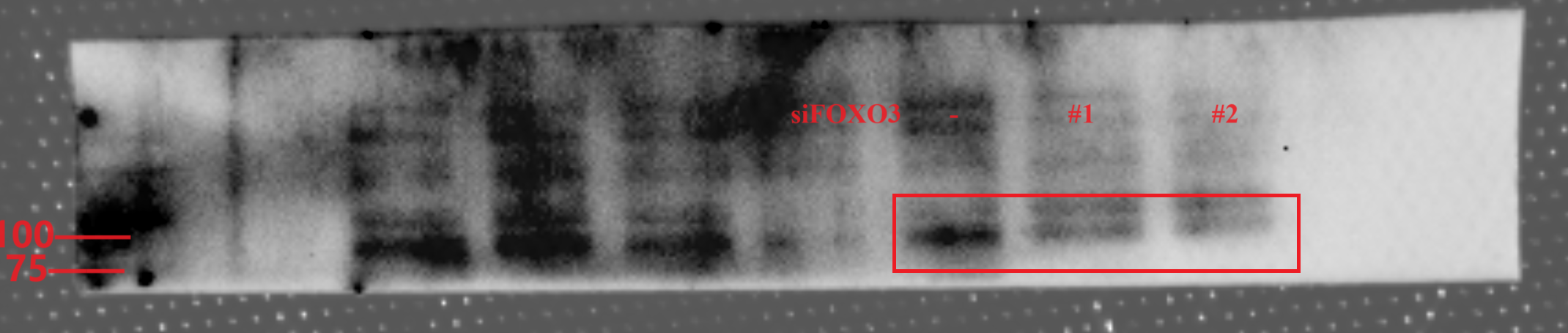

Supplement: Figure 5—source data 1. [file elife-104138-fig5-data1.zip › Figure 5_source Data 1/Figure 5A/5A FOXO3.tif]

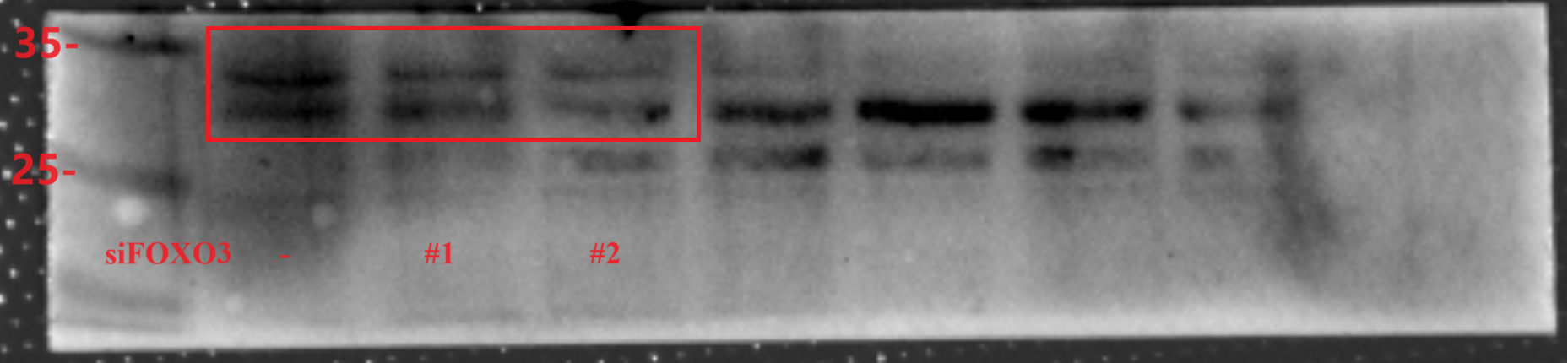

Supplement: Figure 5—source data 1. [file elife-104138-fig5-data1.zip › Figure 5_source Data 1/Figure 5A/5A GNMT.tif]

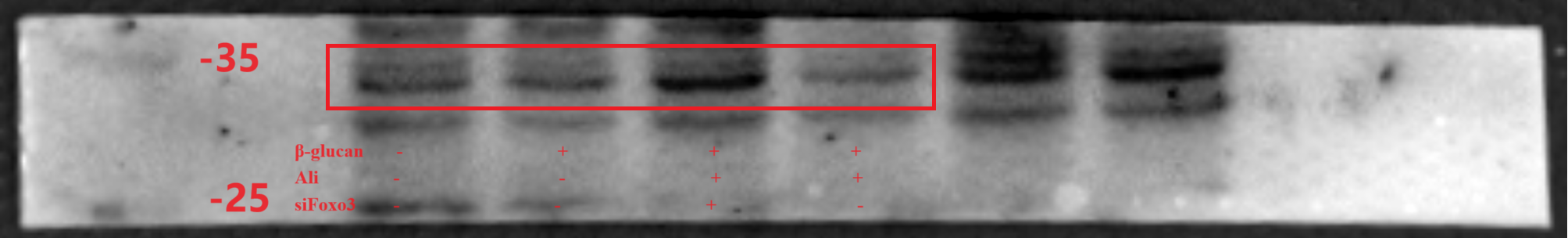

Supplement: Figure 5—source data 1. [file elife-104138-fig5-data1.zip › Figure 5_source Data 1/Figure 5B/5B GNMT.tif]

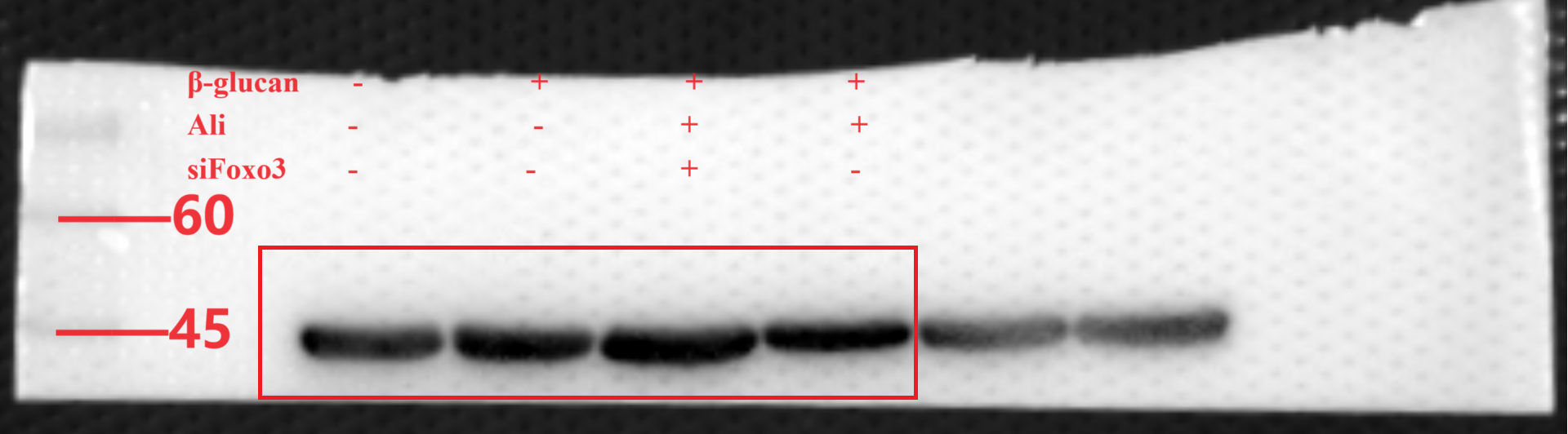

Supplement: Figure 5—source data 1. [file elife-104138-fig5-data1.zip › Figure 5_source Data 1/Figure 5B/5B actin.tif]

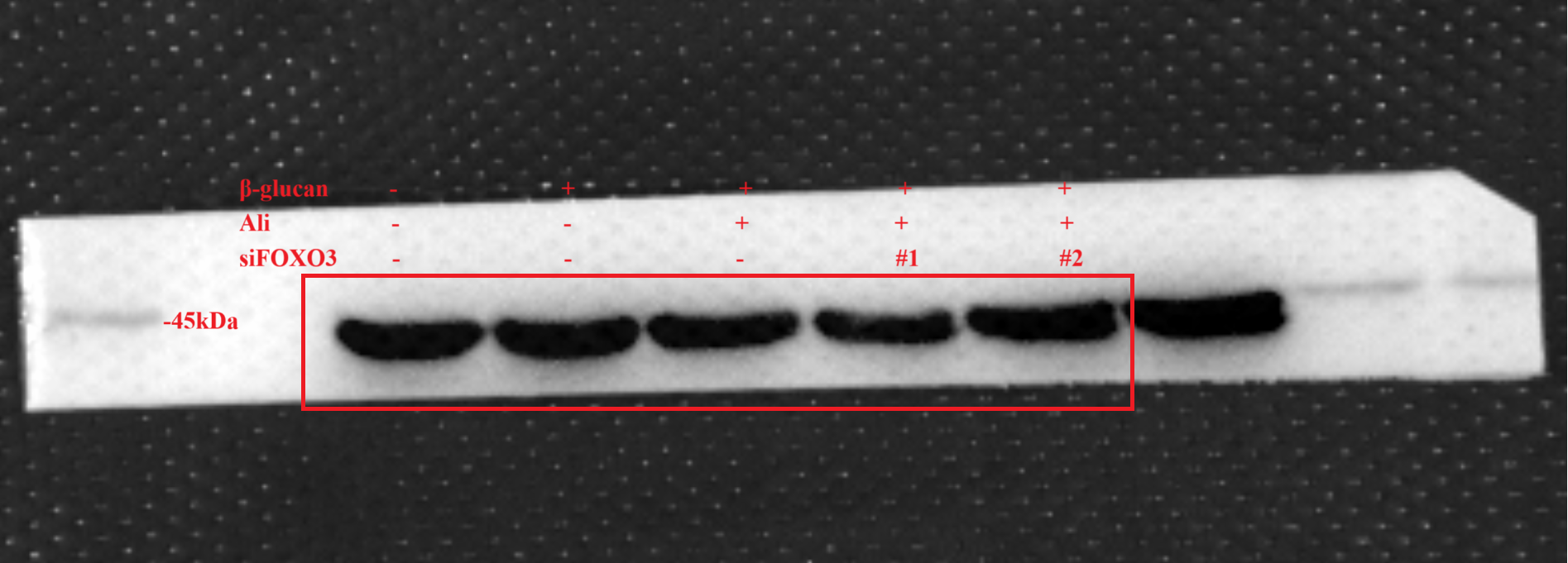

Supplement: Figure 5—source data 1. [file elife-104138-fig5-data1.zip › Figure 5_source Data 1/Figure 5B/rep2/ACTIN.tif]

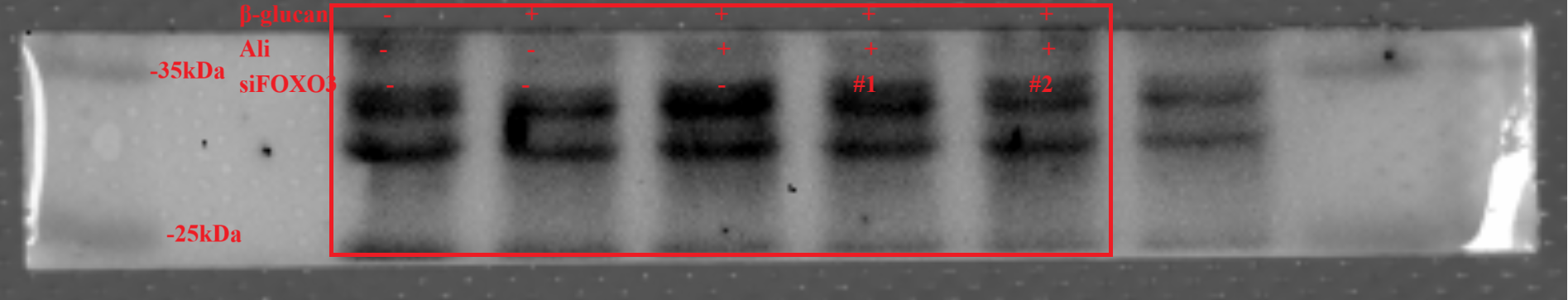

Supplement: Figure 5—source data 1. [file elife-104138-fig5-data1.zip › Figure 5_source Data 1/Figure 5B/rep2/GNMT.tif]

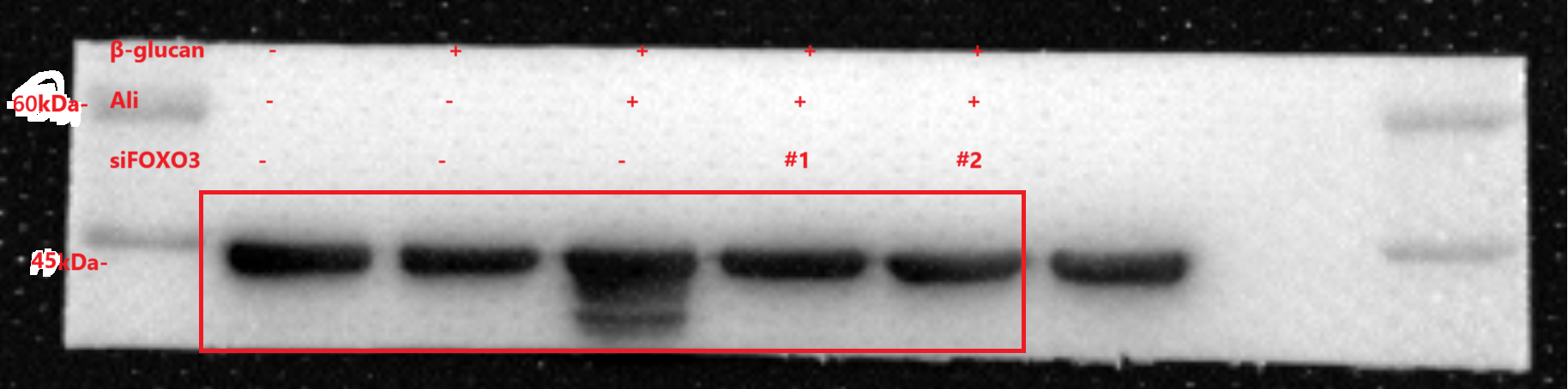

Supplement: Figure 5—source data 1. [file elife-104138-fig5-data1.zip › Figure 5_source Data 1/Figure 5B/rep3/ACTIN.tif]

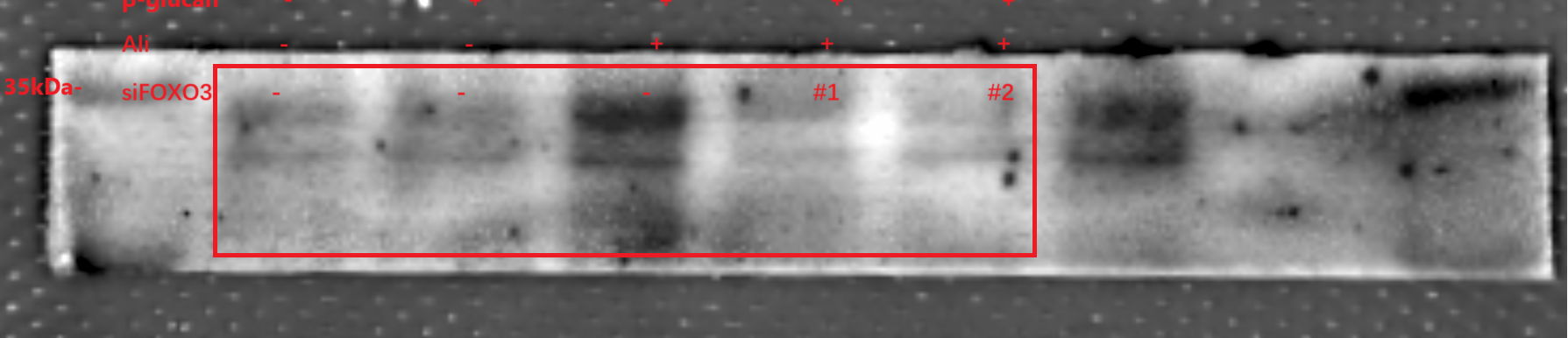

Supplement: Figure 5—source data 1. [file elife-104138-fig5-data1.zip › Figure 5_source Data 1/Figure 5B/rep3/GNMT.tif]

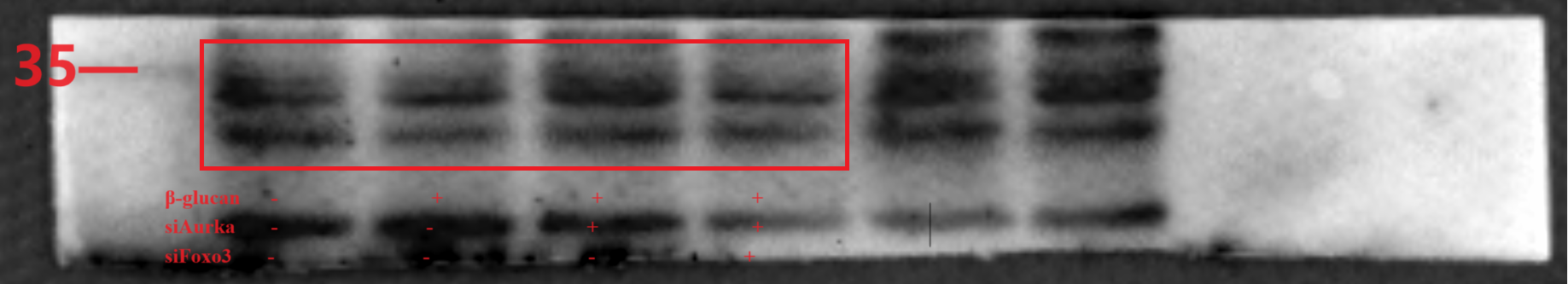

Supplement: Figure 5—source data 1. [file elife-104138-fig5-data1.zip › Figure 5_source Data 1/Figure 5C/5C GNMT.tif]

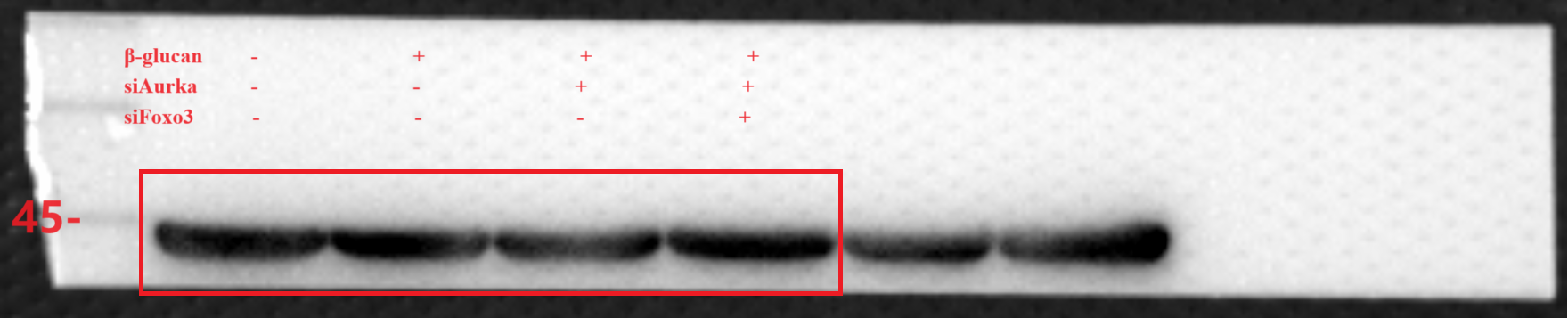

Supplement: Figure 5—source data 1. [file elife-104138-fig5-data1.zip › Figure 5_source Data 1/Figure 5C/5C ACTIN.tif]

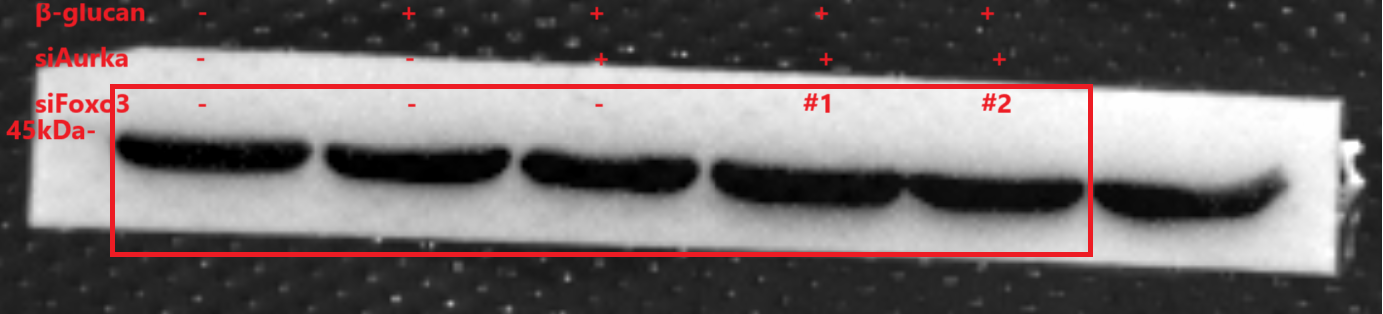

Supplement: Figure 5—source data 1. [file elife-104138-fig5-data1.zip › Figure 5_source Data 1/Figure 5C/rep2/ACTIN.tif]

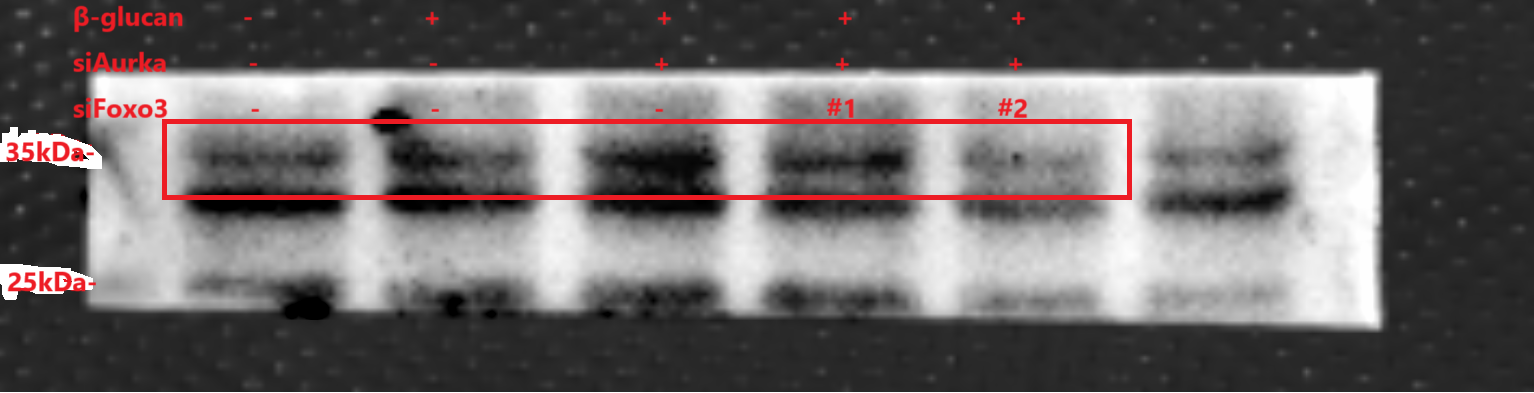

Supplement: Figure 5—source data 1. [file elife-104138-fig5-data1.zip › Figure 5_source Data 1/Figure 5C/rep2/GNMT.tif]

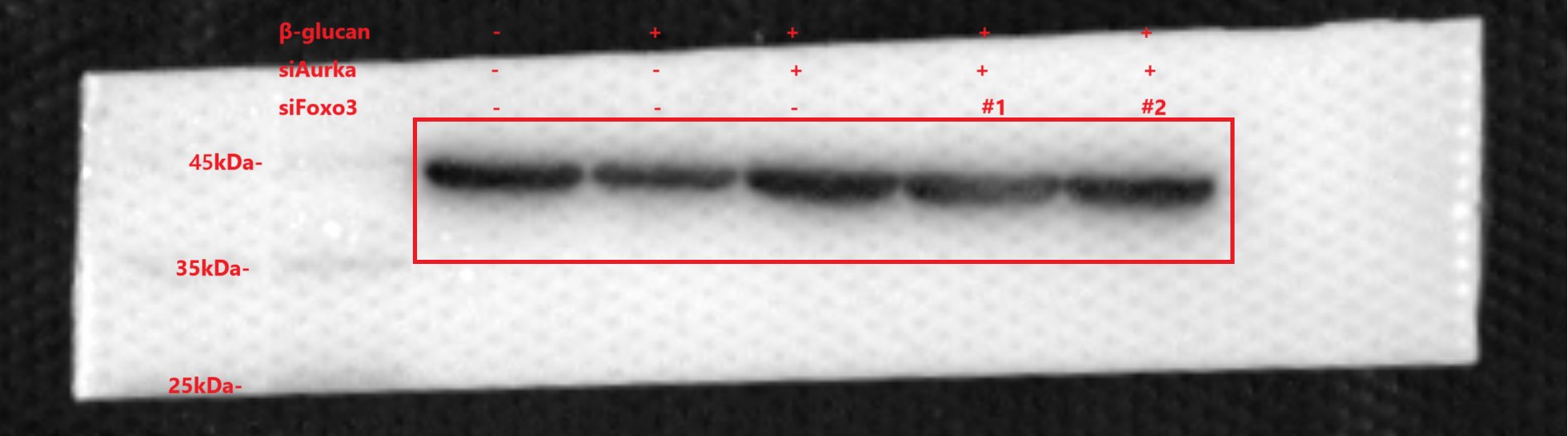

Supplement: Figure 5—source data 1. [file elife-104138-fig5-data1.zip › Figure 5_source Data 1/Figure 5C/rep3/ACTIN.tif]

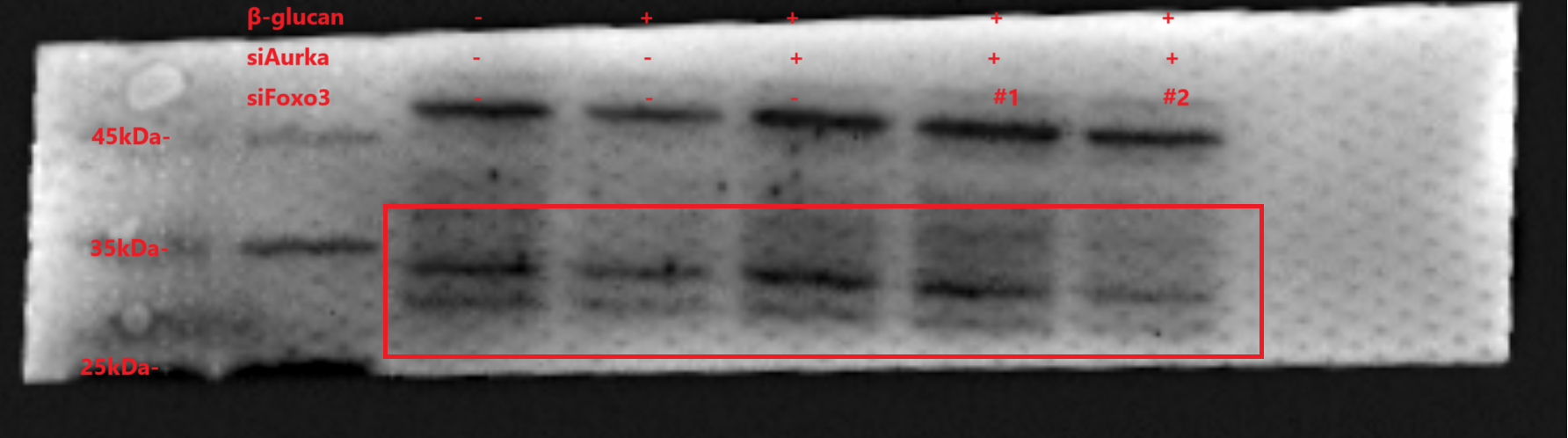

Supplement: Figure 5—source data 1. [file elife-104138-fig5-data1.zip › Figure 5_source Data 1/Figure 5C/rep3/GNMT.tif]

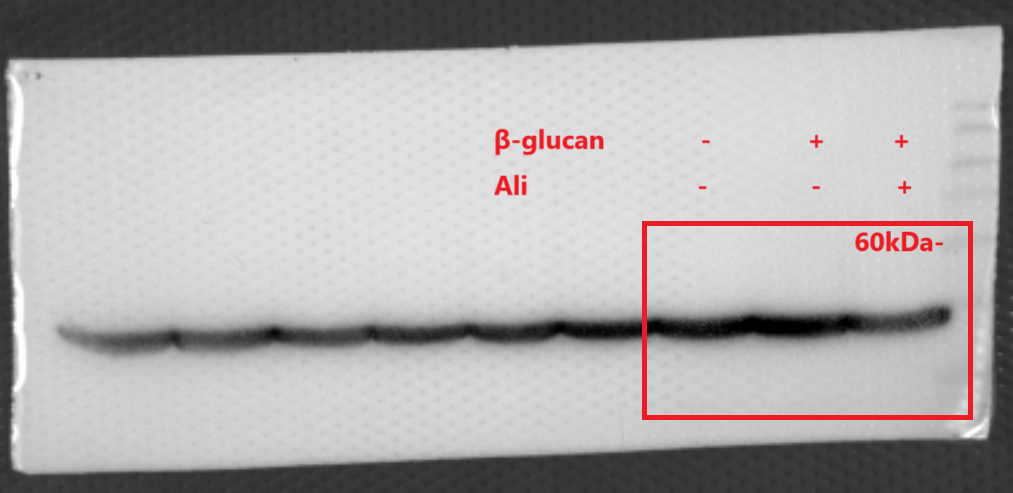

Supplement: Figure 5—source data 1. [file elife-104138-fig5-data1.zip › Figure 5_source Data 1/Figure 5E/5E actin.tif]

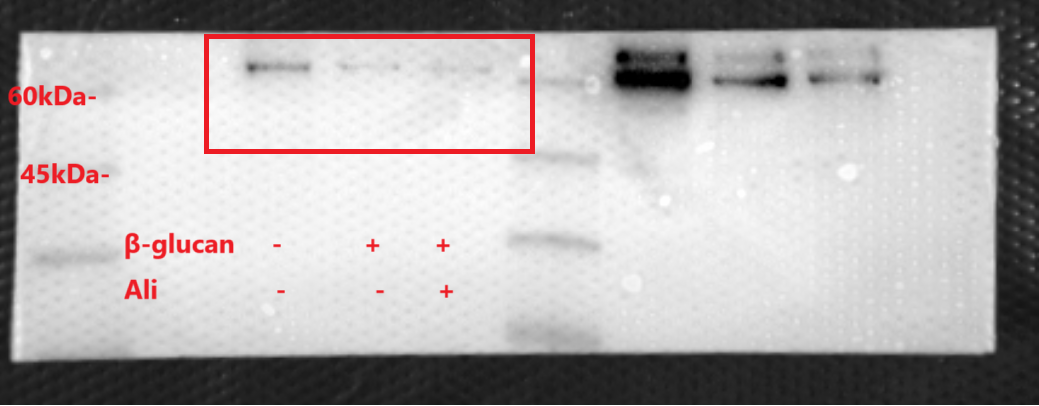

Supplement: Figure 5—source data 1. [file elife-104138-fig5-data1.zip › Figure 5_source Data 1/Figure 5E/5E laminAc.tif]

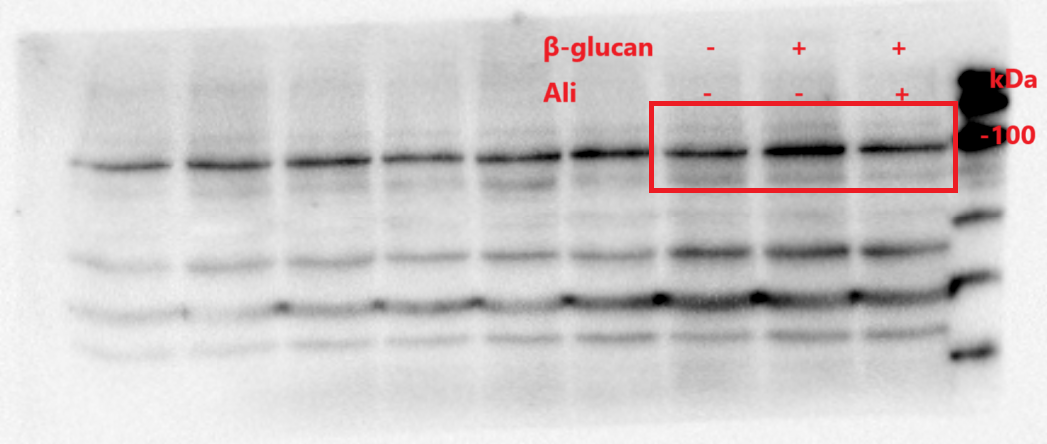

Supplement: Figure 5—source data 1. [file elife-104138-fig5-data1.zip › Figure 5_source Data 1/Figure 5E/5E phospho FOXO3.tif]

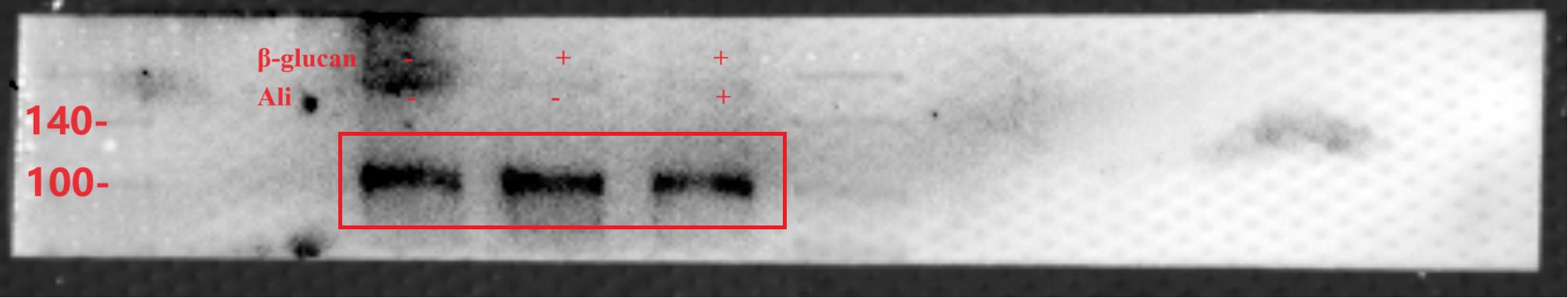

Supplement: Figure 5—source data 1. [file elife-104138-fig5-data1.zip › Figure 5_source Data 1/Figure 5E/5E total FOXO3.tif]

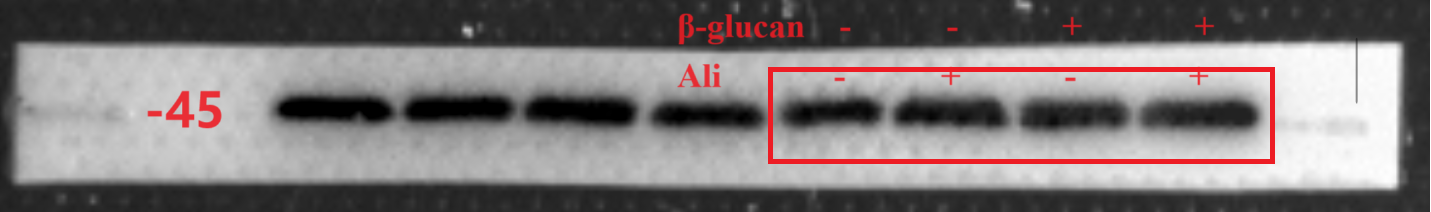

Supplement: Figure 5—source data 1. [file elife-104138-fig5-data1.zip › Figure 5_source Data 1/Figure 5G/5G Ali actin.tif]

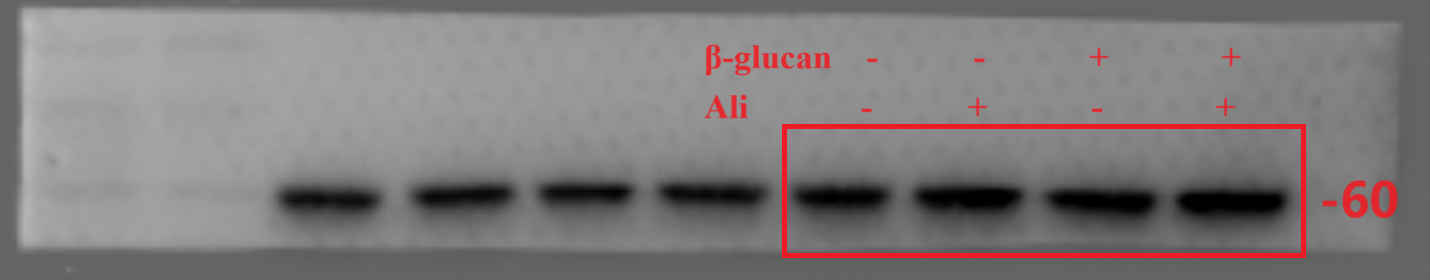

Supplement: Figure 5—source data 1. [file elife-104138-fig5-data1.zip › Figure 5_source Data 1/Figure 5G/5G Ali AKT.tif]

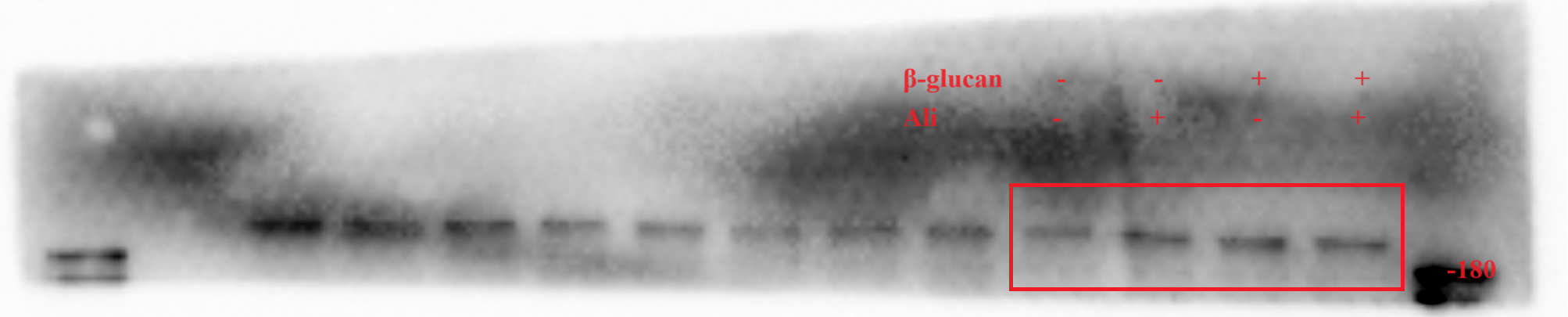

Supplement: Figure 5—source data 1. [file elife-104138-fig5-data1.zip › Figure 5_source Data 1/Figure 5G/5G Ali mTOR.tif]

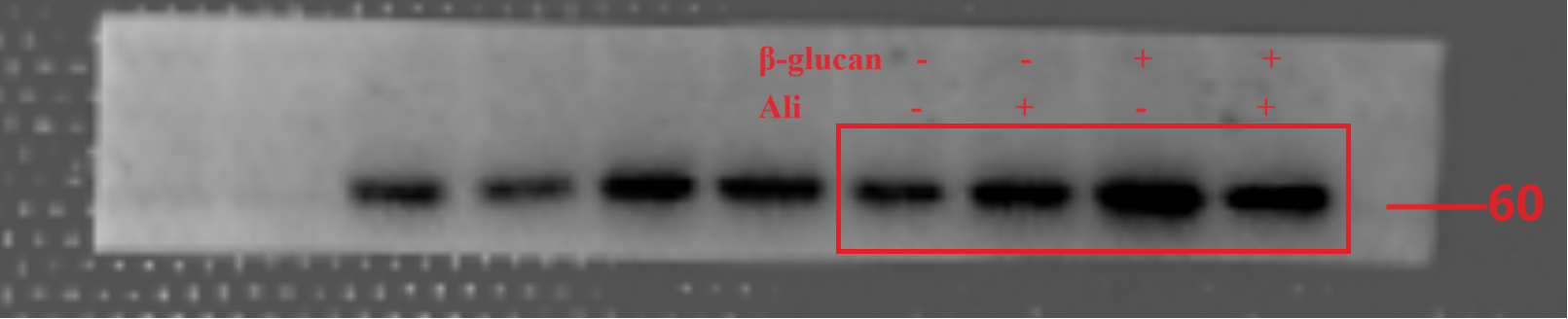

Supplement: Figure 5—source data 1. [file elife-104138-fig5-data1.zip › Figure 5_source Data 1/Figure 5G/5G Ali pAKT.tif]

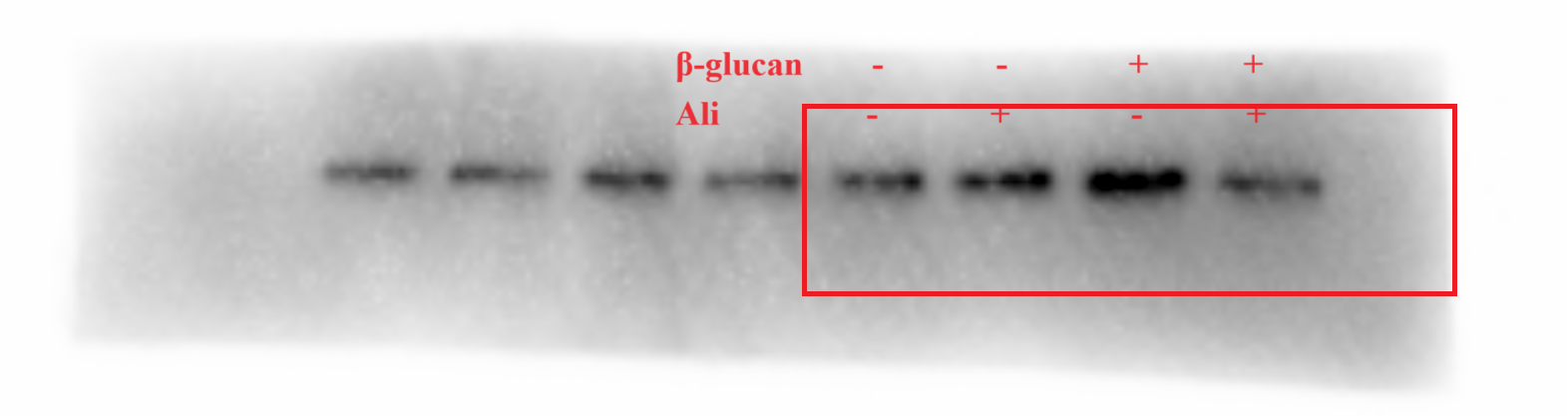

Supplement: Figure 5—source data 1. [file elife-104138-fig5-data1.zip › Figure 5_source Data 1/Figure 5G/5G Ali pmTOR.tif]

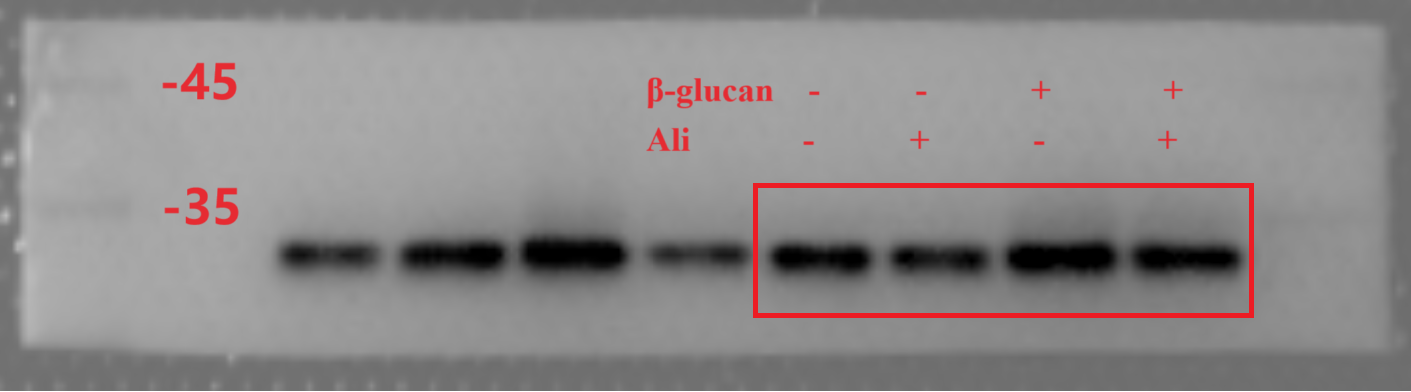

Supplement: Figure 5—source data 1. [file elife-104138-fig5-data1.zip › Figure 5_source Data 1/Figure 5G/5G Ali pS6.tif]

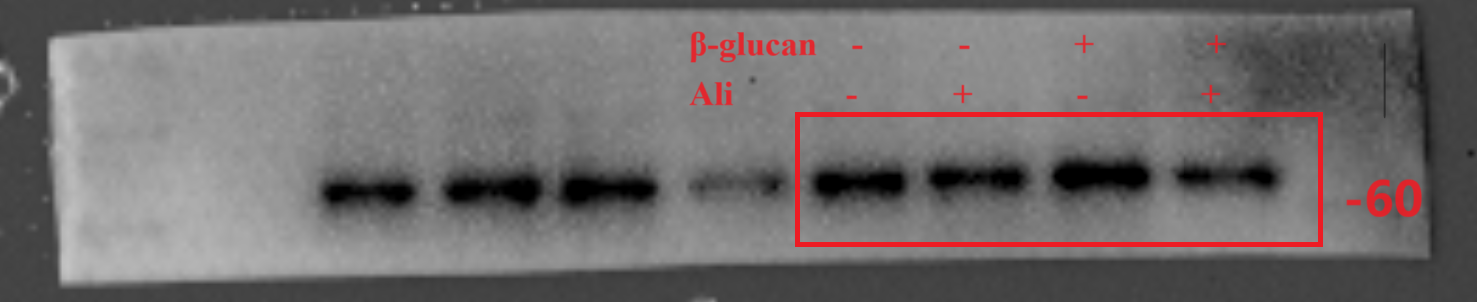

Supplement: Figure 5—source data 1. [file elife-104138-fig5-data1.zip › Figure 5_source Data 1/Figure 5G/5G Ali pS6K.tif]

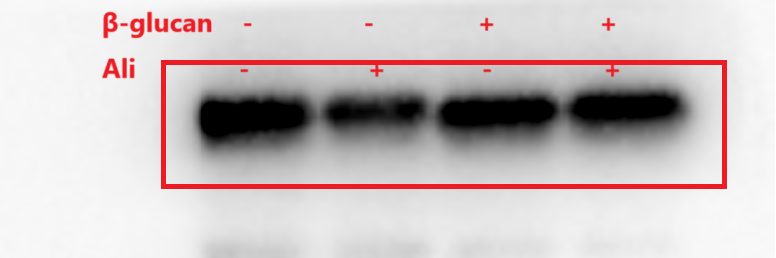

Supplement: Figure 5—source data 1. [file elife-104138-fig5-data1.zip › Figure 5_source Data 1/Figure 5G/5G Ali S6.tif]

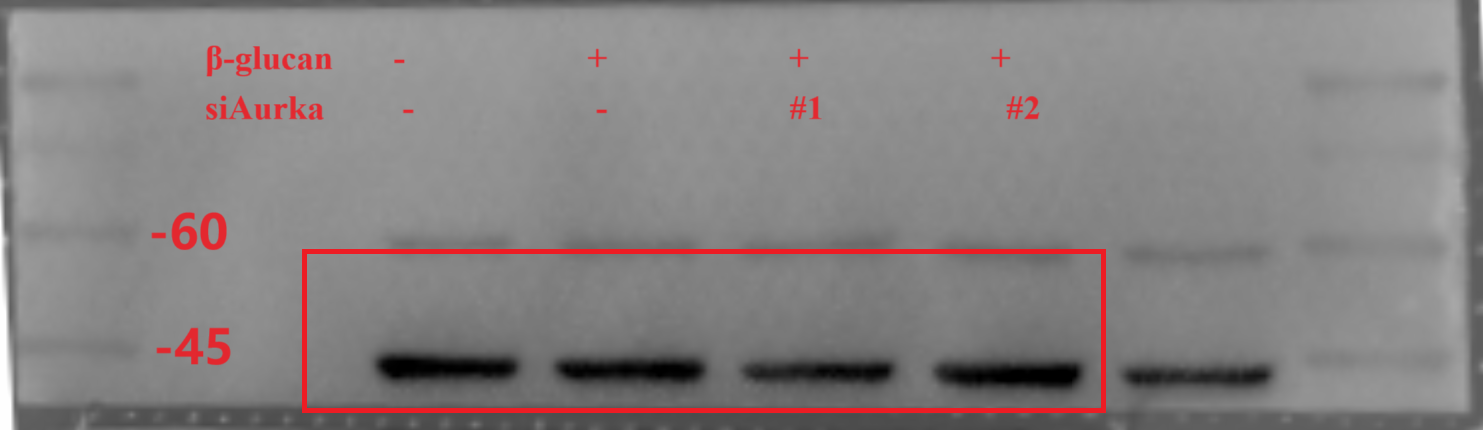

Supplement: Figure 5—source data 1. [file elife-104138-fig5-data1.zip › Figure 5_source Data 1/Figure 5G/5G si actin.tif]

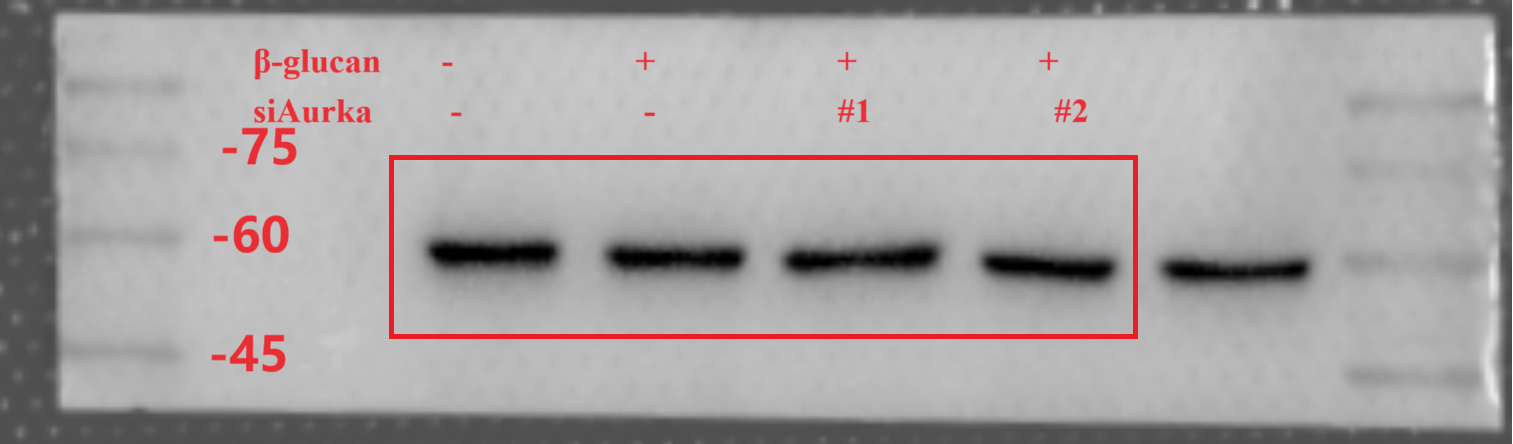

Supplement: Figure 5—source data 1. [file elife-104138-fig5-data1.zip › Figure 5_source Data 1/Figure 5G/5G si AKT.tif]

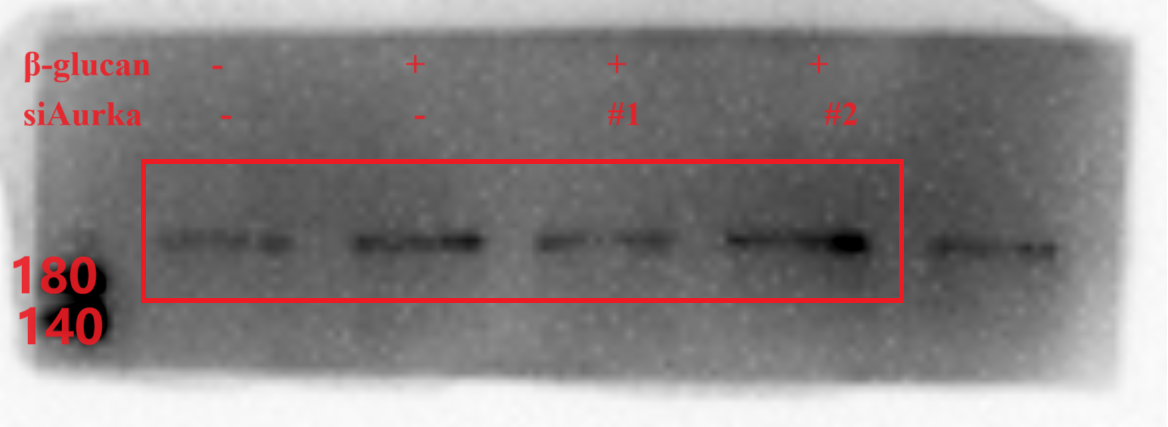

Supplement: Figure 5—source data 1. [file elife-104138-fig5-data1.zip › Figure 5_source Data 1/Figure 5G/5G si mTOR.tif]

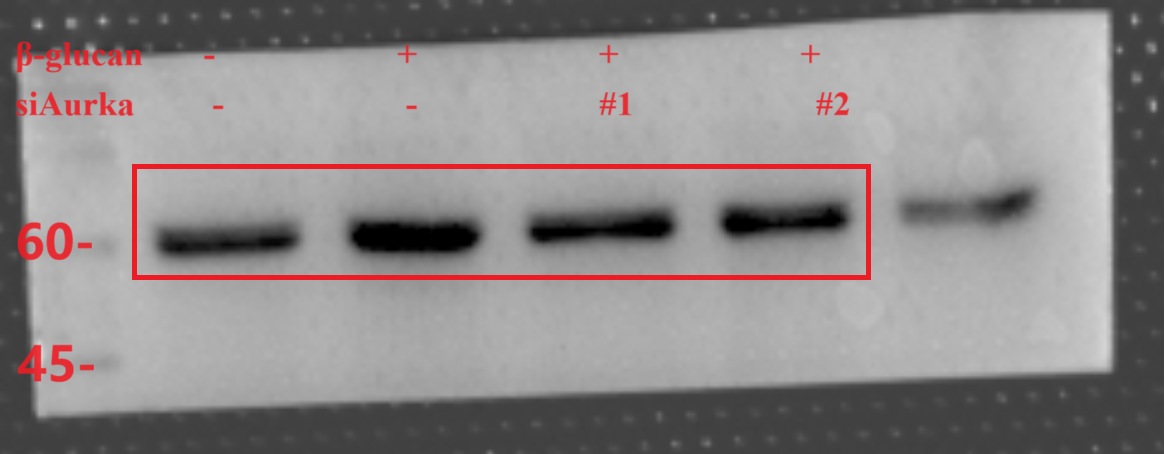

Supplement: Figure 5—source data 1. [file elife-104138-fig5-data1.zip › Figure 5_source Data 1/Figure 5G/5G si pAKT.tif]

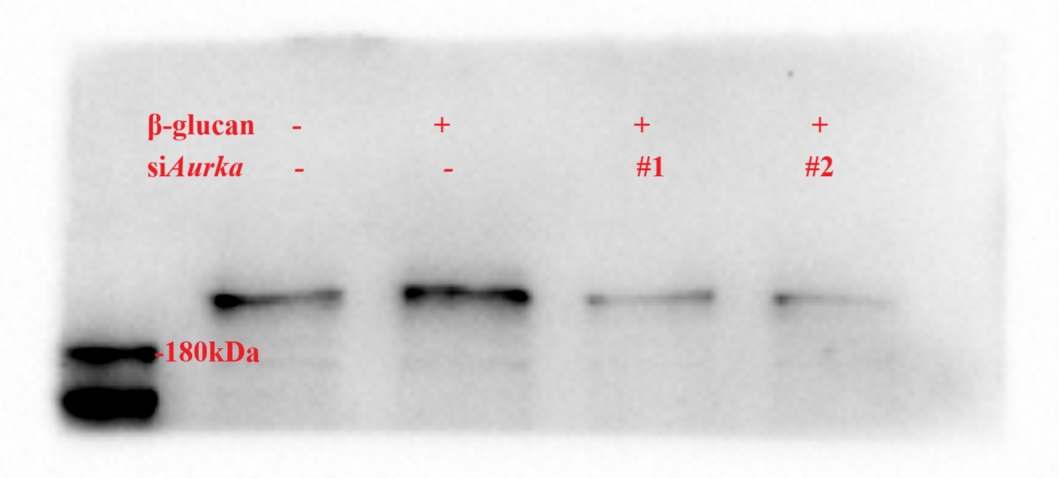

Supplement: Figure 5—source data 1. [file elife-104138-fig5-data1.zip › Figure 5_source Data 1/Figure 5G/5G si pMtor.tif]

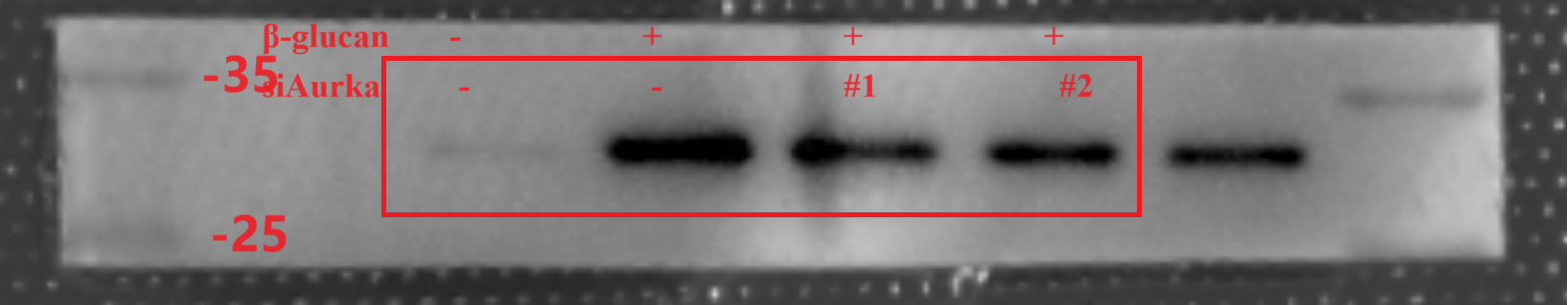

Supplement: Figure 5—source data 1. [file elife-104138-fig5-data1.zip › Figure 5_source Data 1/Figure 5G/5G si pS6.tif]

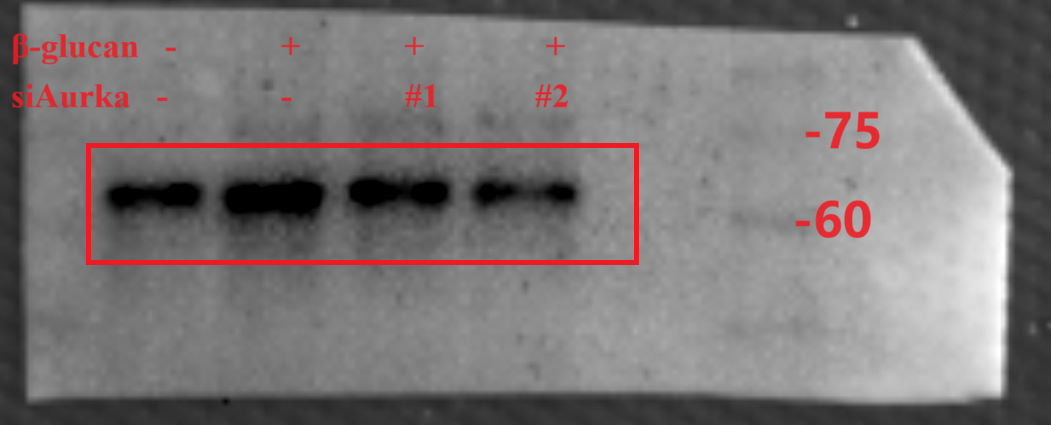

Supplement: Figure 5—source data 1. [file elife-104138-fig5-data1.zip › Figure 5_source Data 1/Figure 5G/5G si pS6K.tif]

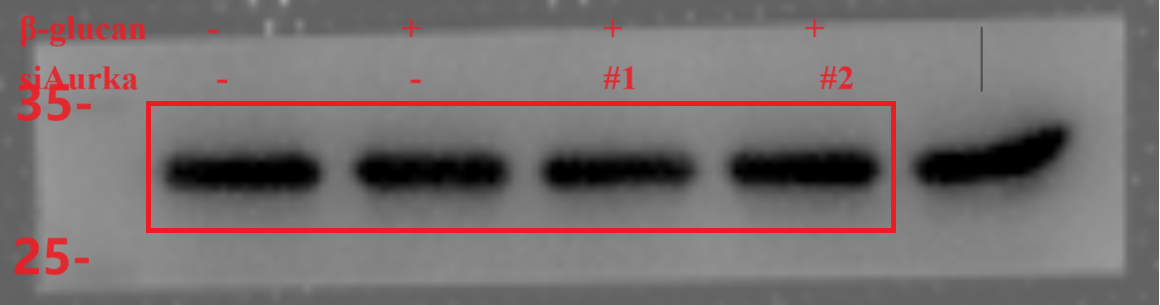

Supplement: Figure 5—source data 1. [file elife-104138-fig5-data1.zip › Figure 5_source Data 1/Figure 5G/5G si S6.tif]

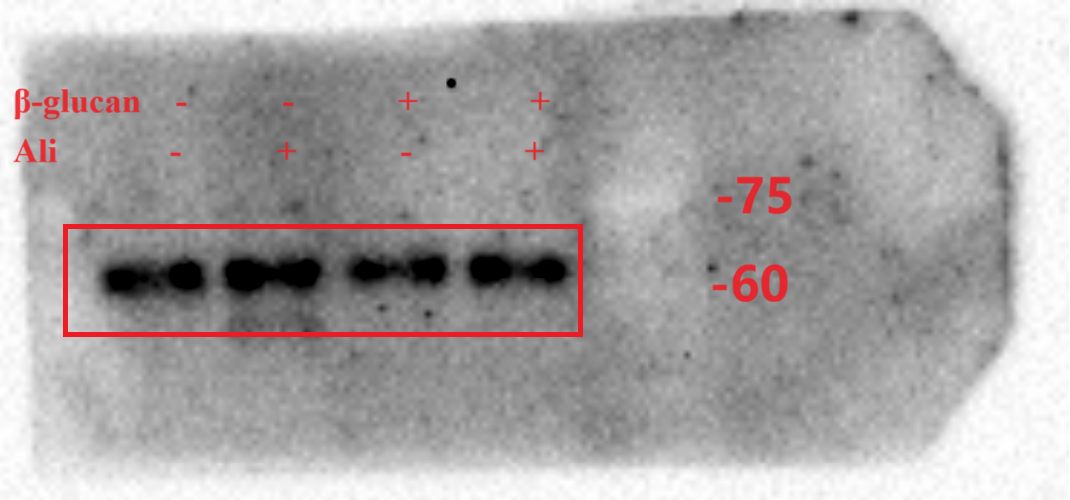

Supplement: Figure 5—source data 1. [file elife-104138-fig5-data1.zip › Figure 5_source Data 1/Figure 5G/Figure 5–source Data 1 Uncropped and labeled blots for 5G Ali S6K.tif]

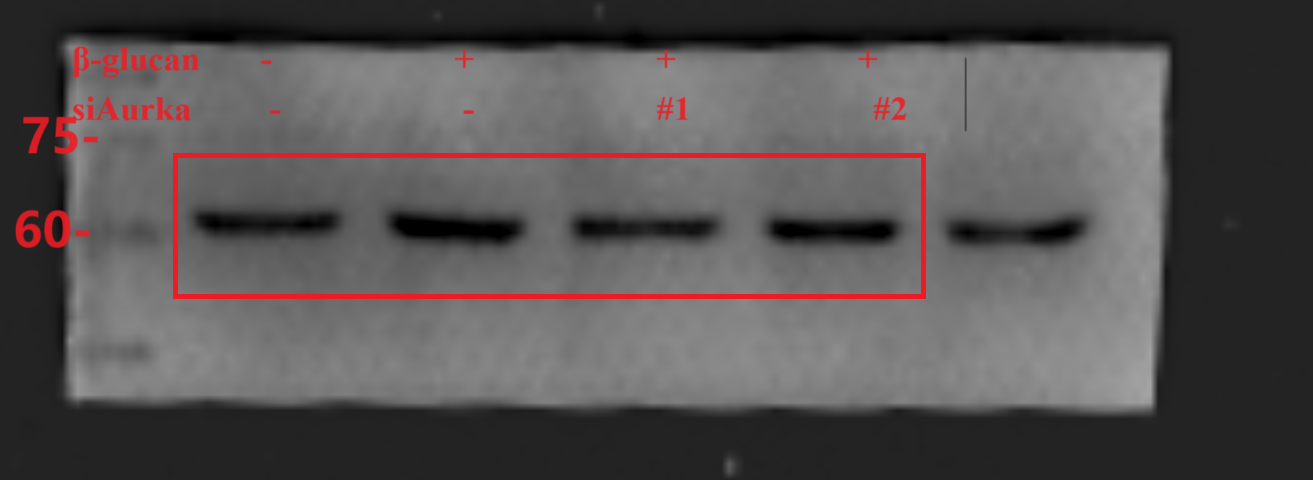

Supplement: Figure 5—source data 1. [file elife-104138-fig5-data1.zip › Figure 5_source Data 1/Figure 5G/Figure 5–source Data 1 Uncropped and labeled blots for 5G si S6K.tif]

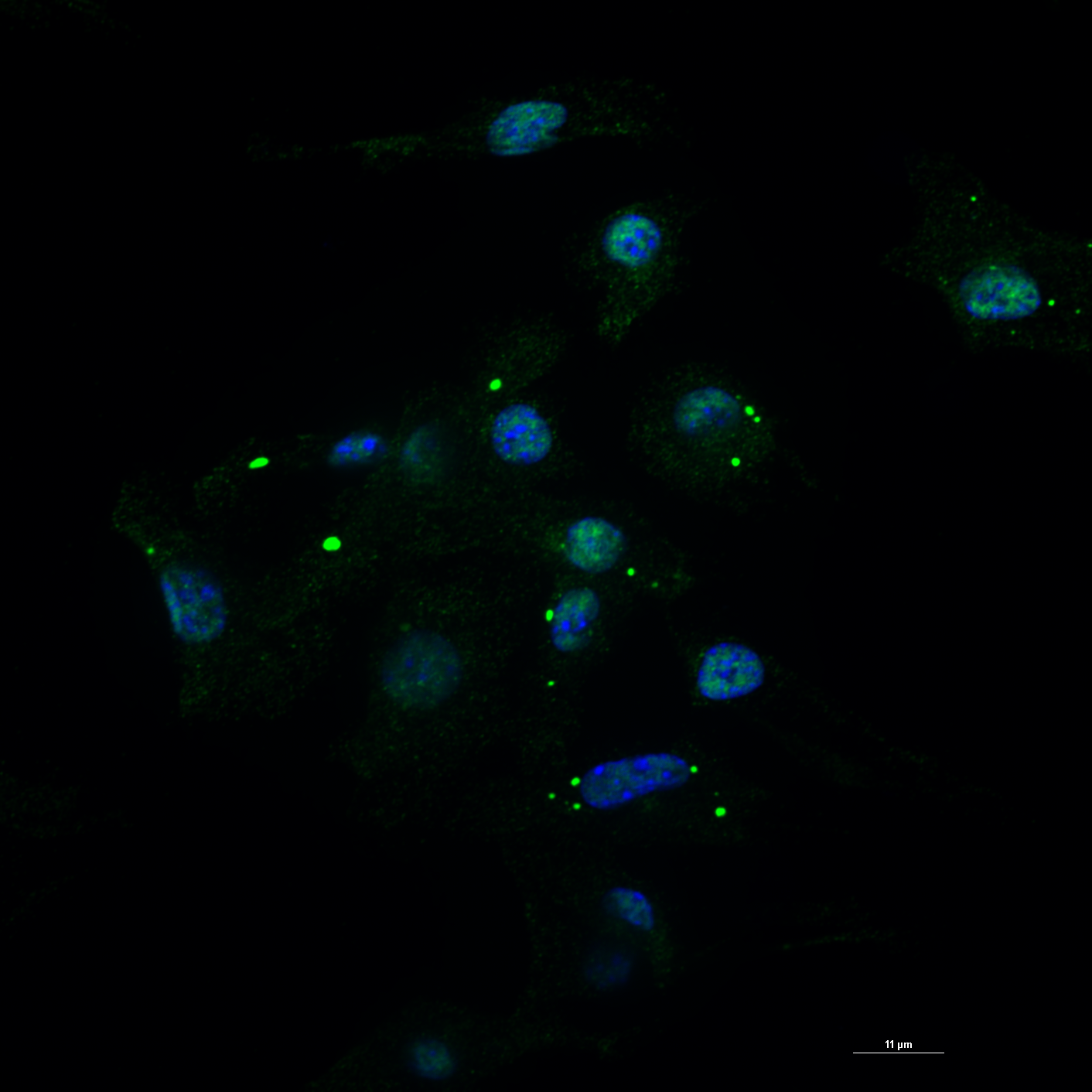

Supplement: Figure 5—source data 3. [file elife-104138-fig5-data3.zip › Figure 5_source Data 3/glucan/b 003_RGB.tif]

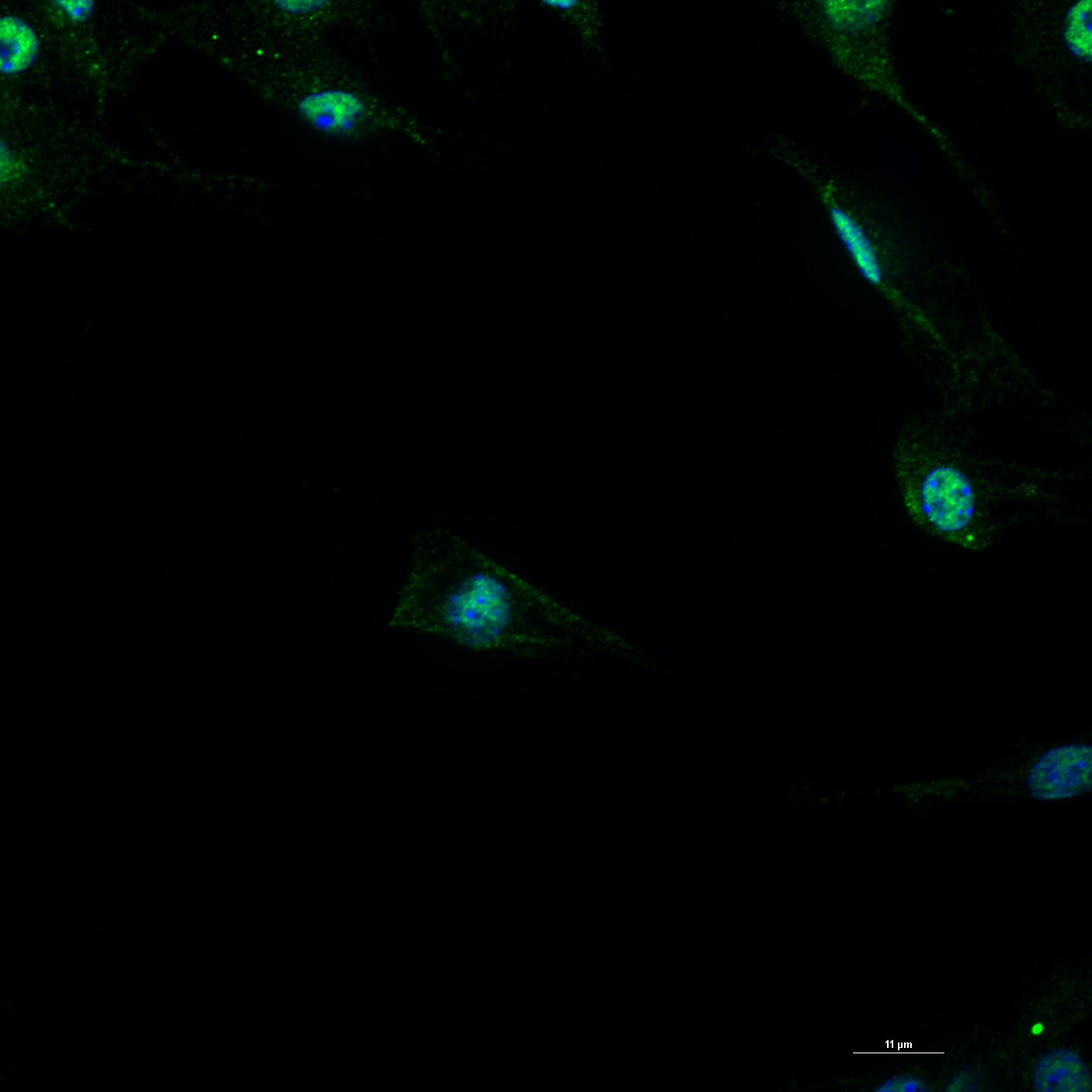

Supplement: Figure 5—source data 3. [file elife-104138-fig5-data3.zip › Figure 5_source Data 3/glucan/b 004_RGB.tif]

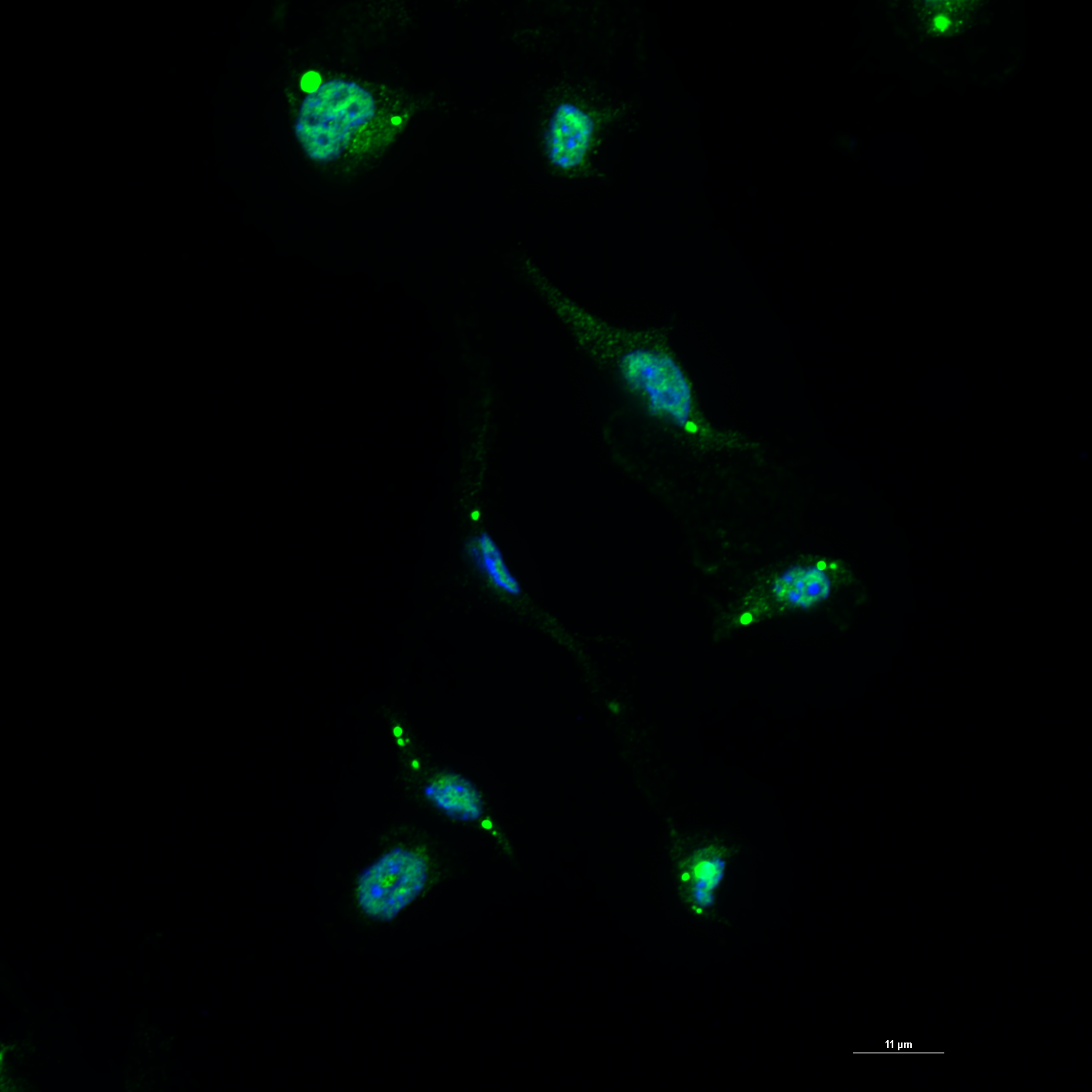

Supplement: Figure 5—source data 3. [file elife-104138-fig5-data3.zip › Figure 5_source Data 3/glucan/b 008_RGB.tif]

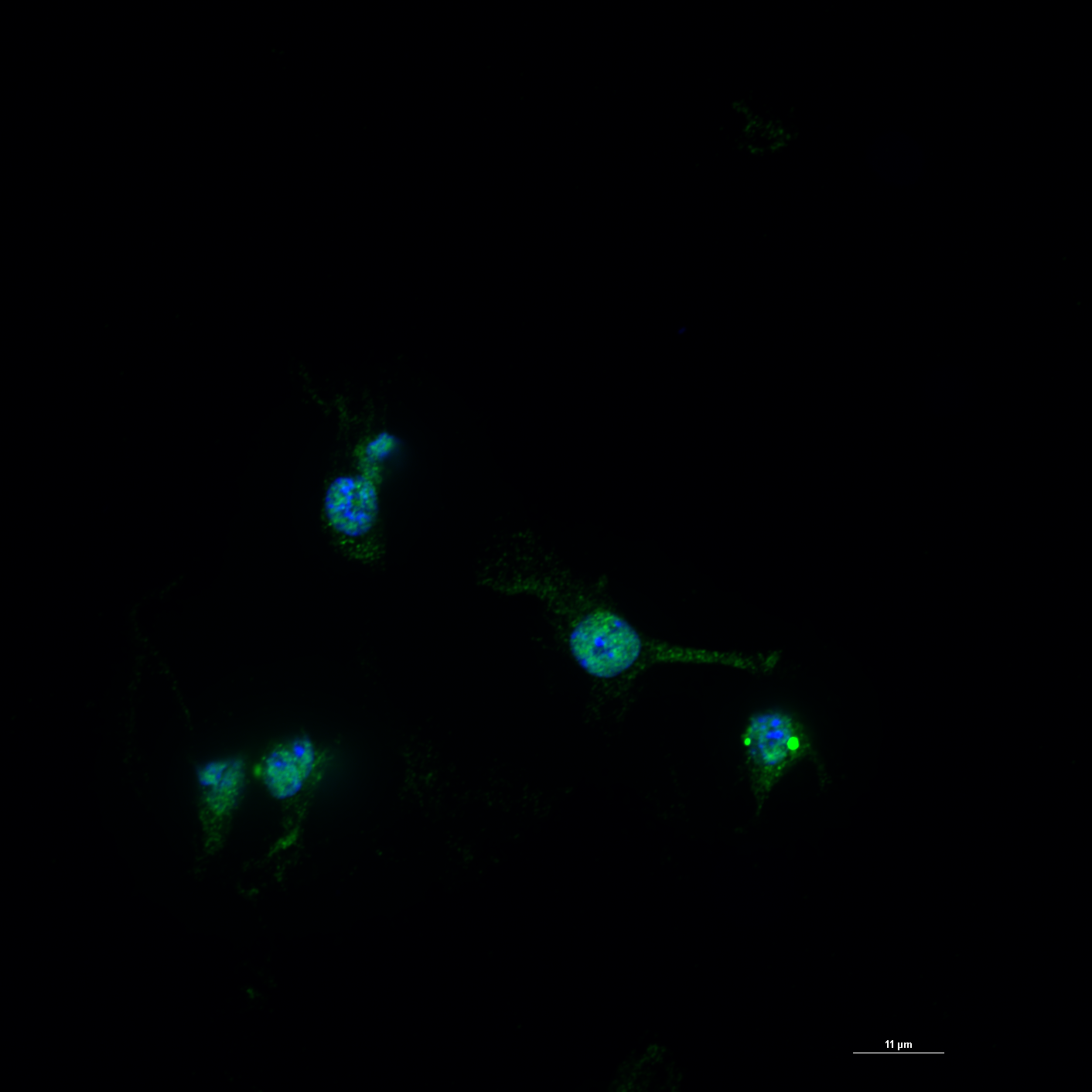

Supplement: Figure 5—source data 3. [file elife-104138-fig5-data3.zip › Figure 5_source Data 3/glucan/b 009_RGB.tif]

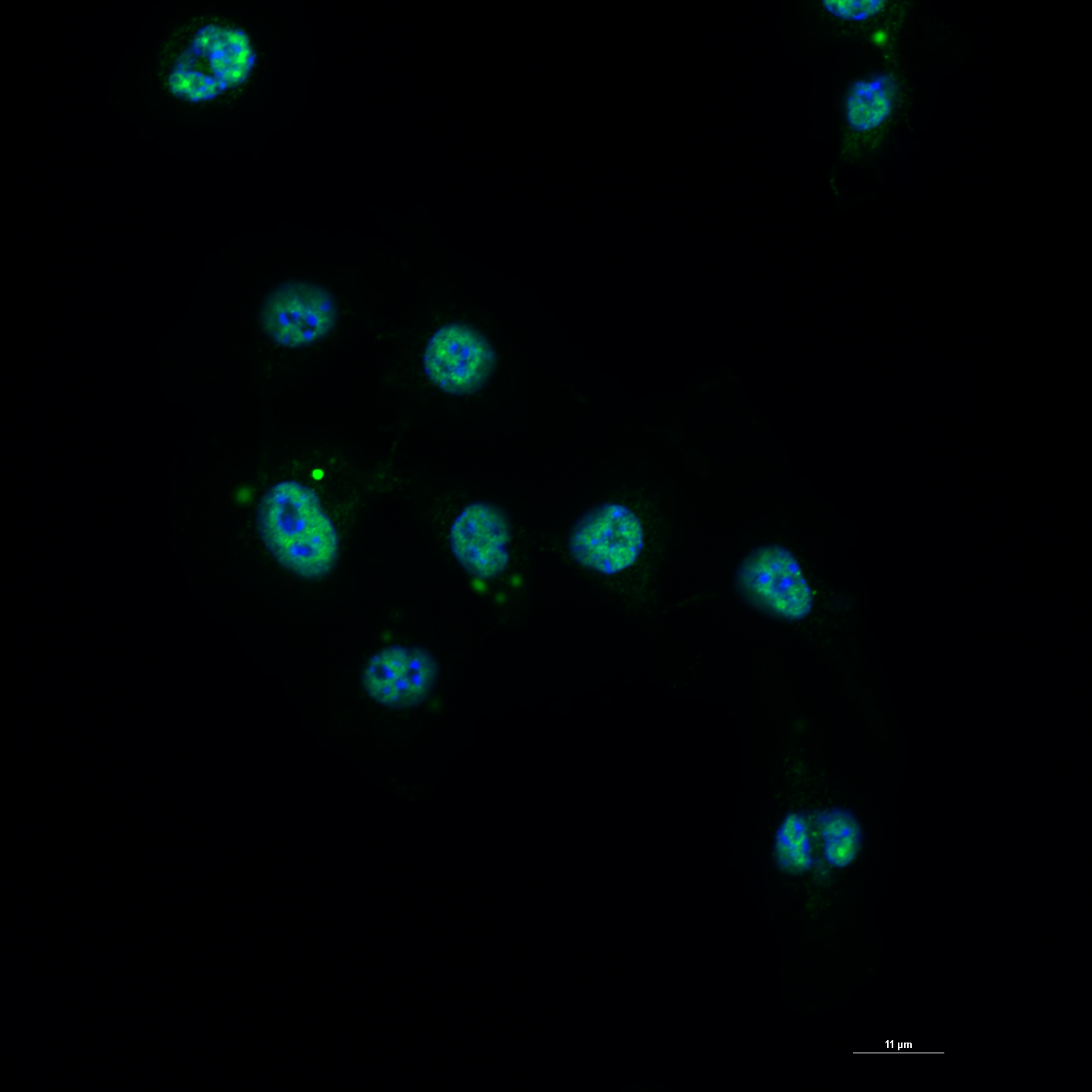

Supplement: Figure 5—source data 3. [file elife-104138-fig5-data3.zip › Figure 5_source Data 3/glucan_Ali/ba 002_RGB.tif]

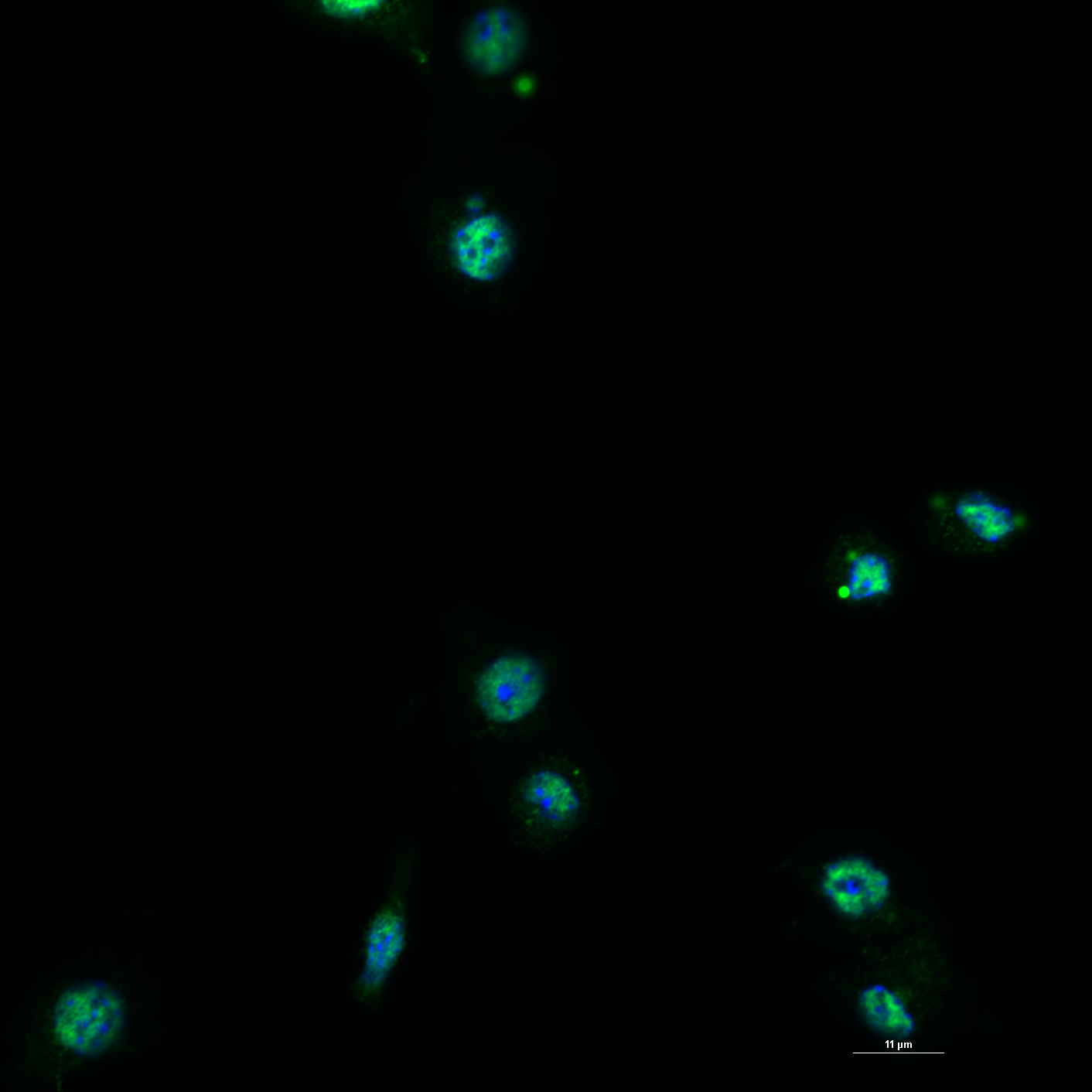

Supplement: Figure 5—source data 3. [file elife-104138-fig5-data3.zip › Figure 5_source Data 3/glucan_Ali/ba 004_RGB.tif]

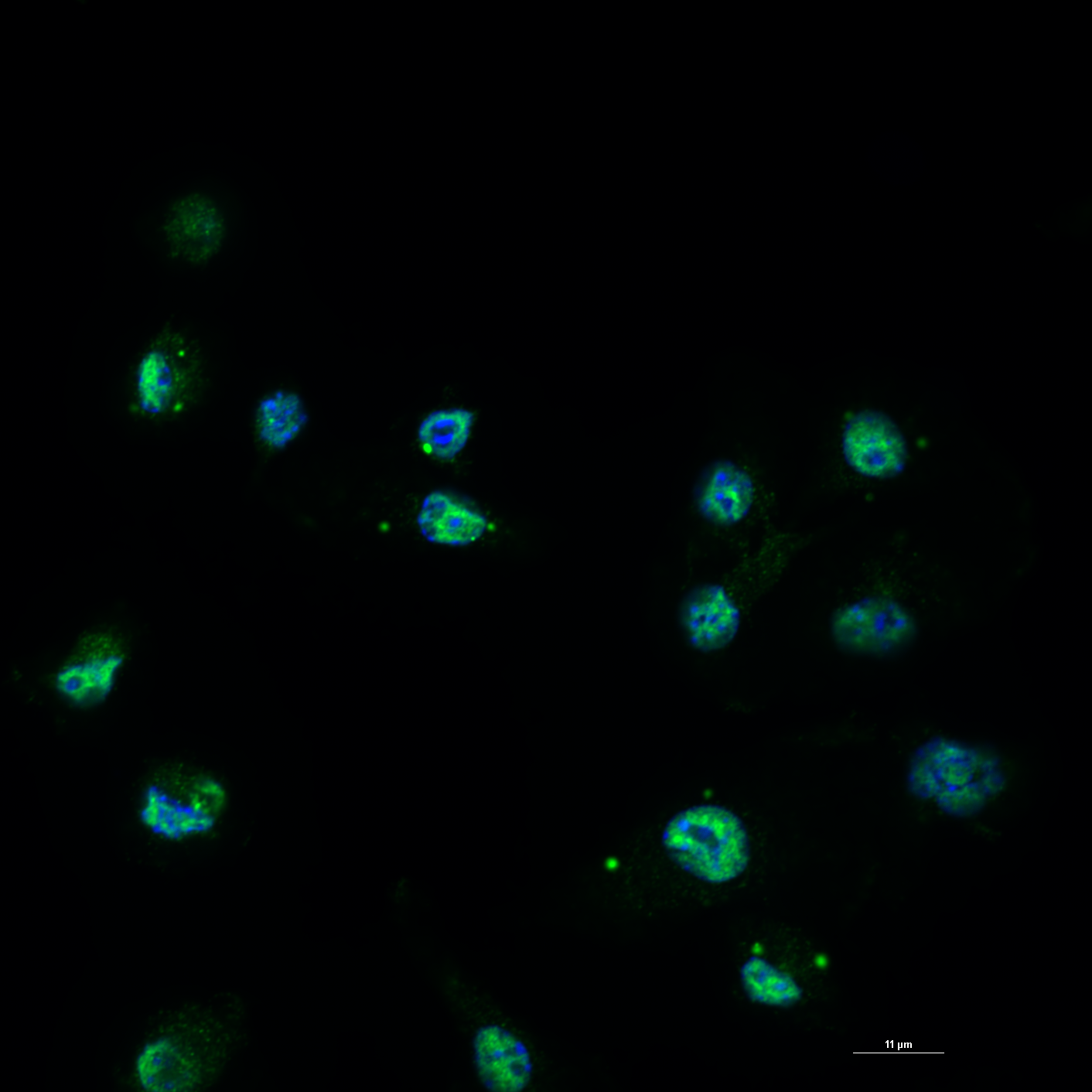

Supplement: Figure 5—source data 3. [file elife-104138-fig5-data3.zip › Figure 5_source Data 3/glucan_Ali/ba 006_RGB.tif]

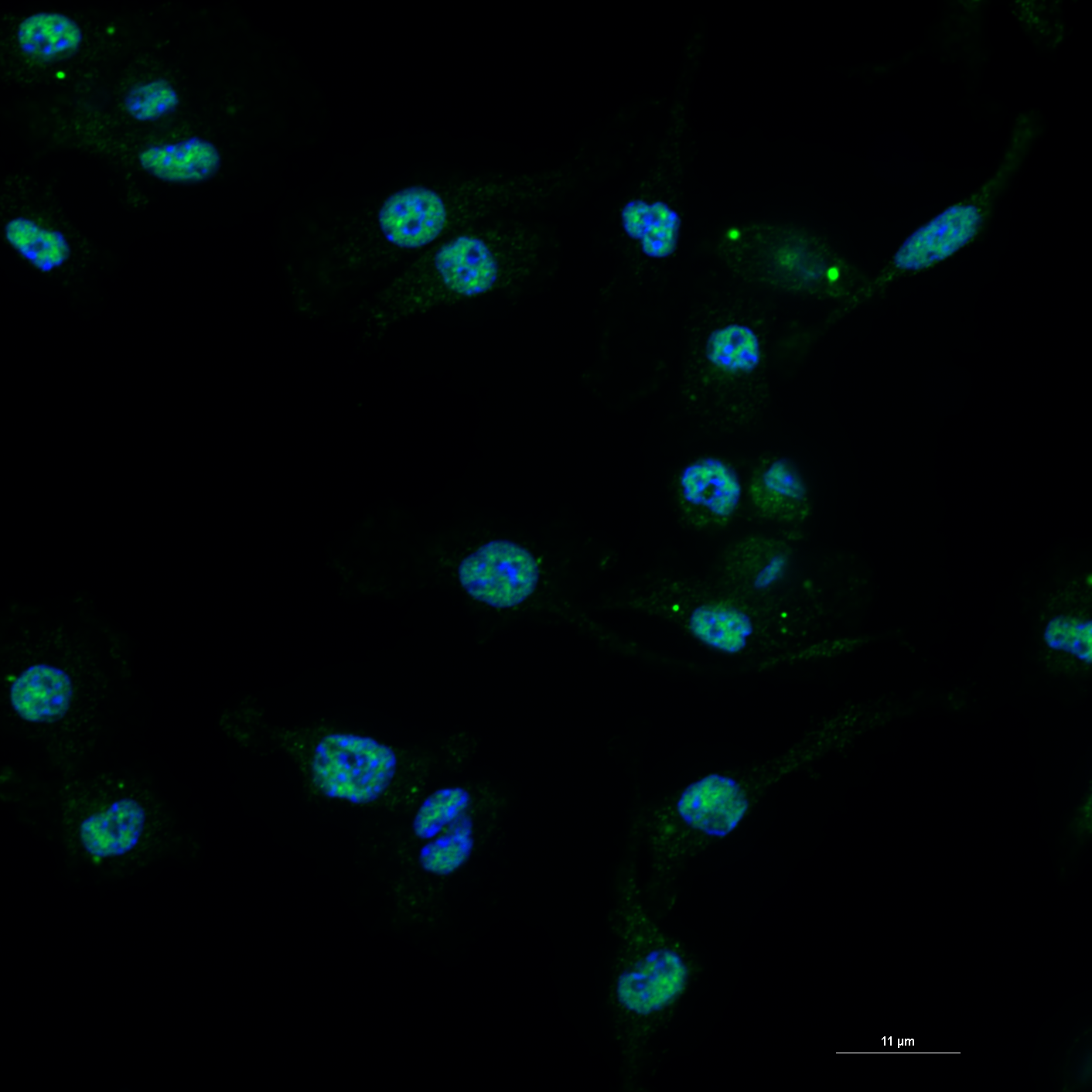

Supplement: Figure 5—source data 3. [file elife-104138-fig5-data3.zip › Figure 5_source Data 3/mock/m 003_RGB.tif]

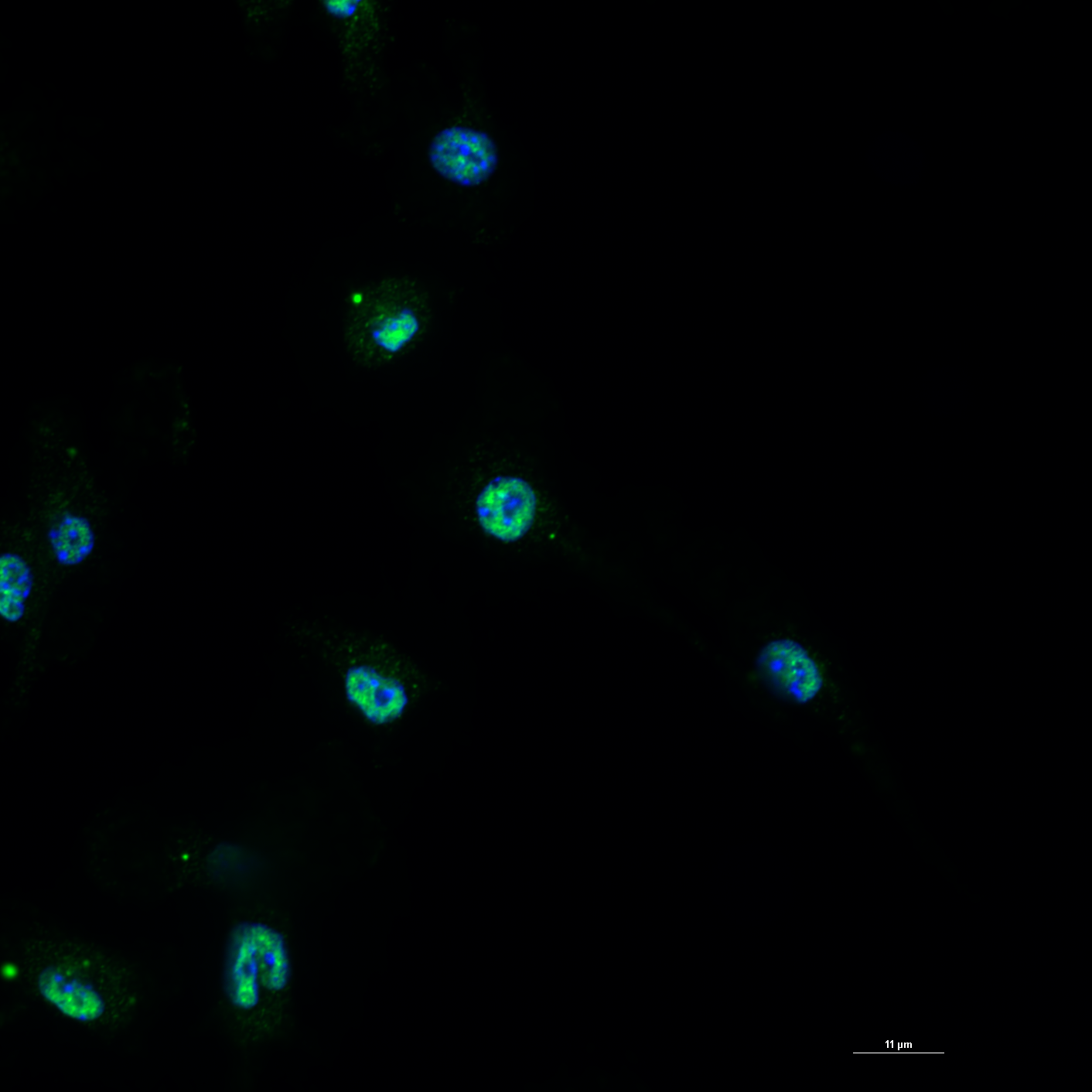

Supplement: Figure 5—source data 3. [file elife-104138-fig5-data3.zip › Figure 5_source Data 3/mock/m 011_RGB.tif]

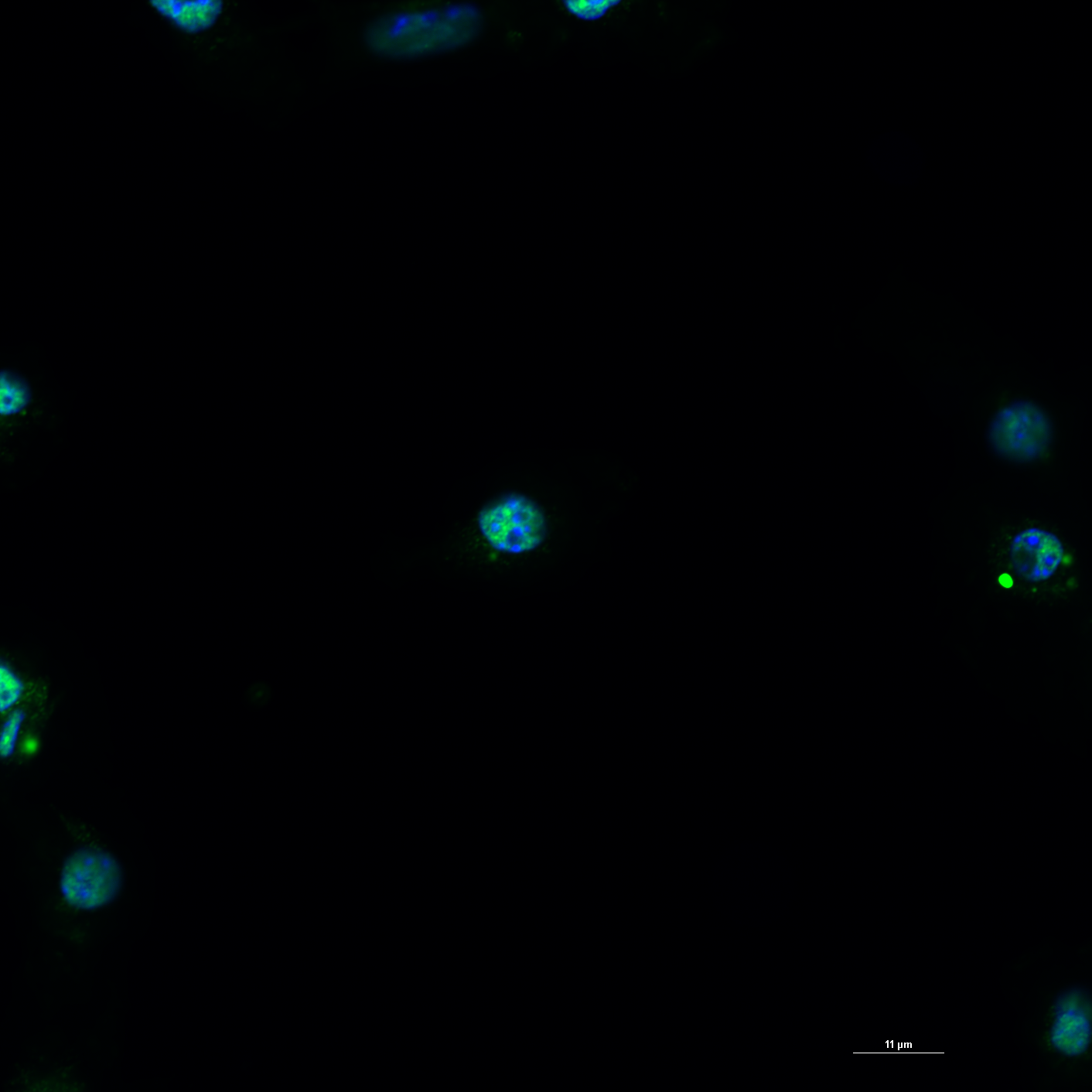

Supplement: Figure 5—source data 3. [file elife-104138-fig5-data3.zip › Figure 5_source Data 3/mock/m 014_RGB.tif]
